# Density Functional Theory for Molecular and Periodic Systems in TURBOMOLE: Theory, Implementation, and Applications

**DOI:** 10.1021/acs.jpca.5c02937

**Published:** 2025-09-23

**Authors:** Manas Sharma, Yannick J. Franzke, Christof Holzer, Fabian Pauly, Marek Sierka

**Affiliations:** † Otto Schott Institute of Materials Research, 9378 Friedrich Schiller University Jena, Löbdergraben 32, 07743 Jena, Germany; ‡ Department of Chemical Engineering, 28335 Indian Institute of Science, Bengaluru, Karnataka 560012, India; § Institute of Nanotechnology, Karlsruhe Institute of Technology (KIT), Kaiserstr. 12, 76131 Karlsruhe, Germany; ∥ Institute of Theoretical Solid State Physics, 26522 Karlsruhe Institute of Technology (KIT), Wolfgang-Gaede-Str. 1, 76131 Karlsruhe, Germany; ⊥ Insitute for Quantum Materials and Technologies, Karlsruhe Institute of Technology (KIT), Kaiserstr. 12, 76131 Karlsruhe, Germany; # Institute of Physics and Center for Advanced Analytics and Predictive Sciences, University of Augsburg, Universitätsstr. 1, 86159 Augsburg, Germany

## Abstract

This work provides a detailed overview of density functional theory (DFT) methods for treating molecular and periodic systems within the TURBOMOLE software package. The implementation employs Gaussian-type orbitals and is based on efficient real-space techniques and density-fitting approaches for Coulomb interactions. Recent developments are reviewed, including the treatment of relativistic effects with effective core potentials, the incorporation of spin–orbit coupling via two-component formalisms, and the extension to real-time time-dependent DFT (RT-TDDFT). Embedding schemes based on frozen-density and projection-based approaches are also discussed, enabling the combination of DFT with high-level correlated wave function methods and many-body perturbation theory for selected subsystems. Representative applications demonstrate the capabilities across bulk materials, surfaces, low-dimensional nanostructures, and adsorption processes. Additionally, a web-based graphical interface has been developed to support input generation, structure manipulation, and output analysis. By consolidating theoretical foundations, implementation strategies, and application examples, this work provides a reference for the use of periodic DFT methods in quantum chemical and materials science studies.

## Graphical abstract

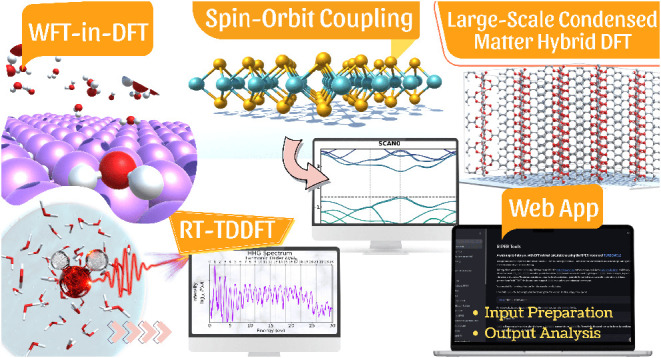

## Introduction

Over the past few decades, density functional theory (DFT) has matured into one of the most powerful and versatile approaches for predicting and rationalizing the electronic structure of atoms, molecules, and extended systems.^1–12^ Its balance of relatively low computational cost with often remarkable accuracy has made DFT a “workhorse” for a wide range of chemical, physical, and materials science problems—from describing molecular reactivity to predicting the properties of bulk solids and surfaces. Advancements in both hardware (high-performance computing, HPC) and software (algorithmic optimizations) have further propelled the field, enabling ever-larger and more complex systems to be studied with increasing precision.

Within this broad landscape, the TURBOMOLE software package^12–14^ has established itself as a highly efficient and robust platform for quantum chemical calculations. Initially recognized for its accuracy and speed in molecular (i.e., finite) DFT and post-Hartree–Fock calculations, it has continuously evolved to include comprehensive capabilities for treating extended (periodic) systems. Central to this development is the Riper module, which provides an efficient and general implementation of periodic DFT employing Gaussian-type orbitals (GTOs). This makes it possible to investigate diverse classes of materials (crystalline solids, surfaces, and low-dimensional nanostructures) under a single unified framework.

A prominent feature of this periodic DFT implementation is its reliance on advanced real-space methods and density-fitting techniques, which ensure both numerical accuracy and favorable scaling with the system size. Moreover, the methods are well parallelized and optimized for modern HPC resources, thus bridging the gap between theoretical innovation and practical feasibility. Recent extensions include (i) scalar- and spin–orbit-coupled relativistic treatments via effective-core and two-component formalisms,^15–17^ (ii) real-time time-dependent DFT (RT-TDDFT) for ultrafast electron dynamics,^18,19^ (iii) efficient hybrid-functional and exact-exchange algorithms,^20^ and (iv) embedding approaches—frozen-density embedding (FDE) and projection-based embedding (PbE)—that seamlessly couple high-level wave function or many-body perturbation methods (e.g., *GW*/BSE) to a periodic DFT environment.^21^ Together, these capabilities expand the scope of TURBOMOLE well beyond conventional applications, enabling, for instance, accurate studies of localized excitations, interfacial charge transfer, and strong spin–orbit or correlation phenomena in solids.

The aim of this work is to present a comprehensive overview of the DFT framework for periodic systems in TURBOMOLE, with particular emphasis on the Riper module. We begin by summarizing the theoretical foundations of periodic DFT and its practical realization using GTOs, shedding light on the resolution-of-the-identity approach and the efficient handling of Coulomb and exchange-correlation (XC) terms. We then discuss more advanced topics, including the two-component (2c) formalism for spin–orbit coupling, local embedding schemes that combine molecular and periodic perspectives, as well as real-time extensions that enable studies of nonequilibrium and ultrafast processes. Following these theoretical and methodological details, a variety of case studies illustrate the capabilities of the code—from prototypical inorganic crystals and surface adsorbates to nanomaterials exhibiting strong spin–orbit or correlation effects—underscoring both the accuracy and efficiency of the TURBOMOLE approach.

We hope this review will serve as a useful resource for both new and experienced researchers alike. By highlighting the underlying theory, implementation strategies, and representative applications, we aim to empower users to fully exploit the periodic DFT functionalities in TURBOMOLE, fostering new discoveries in quantum chemistry, materials science, and beyond.

## General Theory

### Periodic DFT Employing Gaussian Basis Functions

The DFT implementation for periodic systems using GTOs^14,20,22–29^ closely follows standard methods used in comparable computational frameworks. Key equations are presented here to provide an overview of the approach.

In a periodic system, translational symmetry implies that each single-particle orbital (ψ_*pσ*_^**k**^(**r**)) can be expressed as a Bloch function, labeled by the band index *p*, spin σ, and wavevector **k** within the Brillouin zone (BZ). In the GTO picture, each Bloch orbital reads

1 $$\psi_{p\sigma }^{k}\left(r\right)=\frac{1}{\sqrt{N_{\mathrm{UC}}}}\sum_{L}e^{ik^{T}L}\sum_{\mu }C_{\mu p\sigma }^{k}\mu_{L}\left(r\right)$$

where μ­(**r** – **R**_μ_ – **L**) ≡ μ_**L**_(**r**) are GTO basis functions centered at atomic positions **R**_μ_ translated by the direct lattice vector **L**, summing over all *N*_UC_ unit cells. In the Kohn–Sham (KS) formalism, the expansion coefficients *C*_*μpσ*_^**k**^ are obtained by solving matrix equations

2 $$F_{\sigma }^{k}C_{\sigma }^{k}=S^{k}C_{\sigma }^{k}\varepsilon_{\sigma }^{k}$$

independently for each **k** in the BZ. In molecular systems, only **L** = **k** = **0** is relevant, and *N*_UC_ equals one. Here, **F**_σ_^**k**^ and **S** ^**k**^ represent the KS and overlap matrices in reciprocal space, respectively, which are obtained from real-space matrices as

3 $$F_{\mu \nu \sigma }^{k}=\sum_{L}e^{ik^{T}L}F_{\mu \nu \sigma }^{L}$$

4 $$S_{\mu \nu }^{k}=\sum_{L}e^{ik^{T}L}S_{\mu \nu }^{L}$$

The elements *F*_*μνσ*_^**L**^ contain contributions from the kinetic energy *T*_*μν*_^**L**^, Coulomb *J*_*μν*_^**L**^, and XC *X*_*μνσ*_^**L**^ matrices, given by

5 $$F_{\mu \nu \sigma }^{L}=T_{\mu \nu }^{L}+J_{\mu \nu }^{L}+X_{\mu \nu \sigma }^{L}$$

The kinetic-energy terms *T*_*μν*_^**L**^ are evaluated identically to the molecular case, and *X*_*μνσ*_^**L**^ values are computed using a hierarchical integration scheme.^26^ Calculating *J*_*μν*_^**L**^ and *X*_*μνσ*_^**L**^ requires the real-space density matrix *D*_*μνσ*_^**L**^, defined as an integral over the BZ, which is evaluated numerically as

6 $$D_{\mu \nu \sigma }^{L}=\frac{1}{V_{k}}\int_{\mathrm{BZ}}D_{\mu \nu \sigma }^{k}e^{ik^{T}L}\mathrm{dk}\approx \frac{1}{V_{k}}\sum_{k}w_{k}D_{\mu \nu \sigma }^{k}e^{ik^{T}L}$$

where *w*_**k**_ are the weights assigned to each *k*-point. Here, *D*_*μνσ*_^**k**^ is the reciprocal-space density matrix, calculated as

7 $$D_{\mu \nu \sigma }^{k}=\sum_{p}f_{p\sigma }^{k}{\left(C_{\mu p\sigma }^{k}\right)}^{*}C_{\nu p\sigma }^{k}$$

At zero Kelvin, occupation numbers *f*_*pσ*_^**k**^ are either zero or one, representing fully occupied or unoccupied states. For metals, however, fractional occupations are used to smooth the Fermi surface and improve the convergence. At finite temperatures, these fractional occupations are computed using either the Fermi–Dirac distribution

8 $$f_{p\sigma }^{k}=\frac{1}{1+e^{\left(\varepsilon_{p\sigma }^{k}-\mu \right)/\left(k_{B}T\right)}}$$

or Gaussian smearing, where occupations are approximated as

9 $$f_{p\sigma }^{k}=\exp\left(-\frac{{\left(\varepsilon_{p\sigma }^{k}-\mu \right)}^{2}}{2\sigma^{2}}\right)$$

where μ is the chemical potential (or Fermi level), *k*_*B*_ is the Boltzmann constant, *T* is the temperature, and σ is the smearing width in the Gaussian approach. These methods provide smooth fractional occupations for states near the Fermi level, enabling faster convergence in metallic systems.

The total energy per unit cell *E* is calculated as

10 $$E=\sum_{\mu \nu L}\sum_{\sigma }D_{\mu \nu \sigma }^{L}T_{\mu \nu }^{L}+J+E_{\mathrm{xc}}$$

where *J* and *E*_xc_ are the Coulomb and the XC energy, respectively.

### Density-Fitting Scheme for the Coulomb Term in Periodic Systems

Due to the long-range nature of Coulomb interactions, density fitting (DF) for periodic systems requires special techniques. TURBOMOLE implements a projection approach as a direct extension of the molecular DF scheme. A full description of this method can be found in refs 22–25 ; only a summary is provided here.

The total (infinite) electron density of the crystal, ρ^cryst^, is expressed as an infinite sum of local densities ρ_**L**_ over cells translated by the vector **L** as

11 $$\rho^{\mathrm{cryst}}=\sum_{L}\rho_{L}$$

with each local density ρ_**L**_ defined by

12 $$\rho_{L}=\sum_{\mu \nu L^{\prime }}\sum_{\sigma }D_{\mu \nu \sigma }^{L\prime }\mu_{L}\nu_{\mathrm{LL}\prime }$$

where ν_**L**_(**r** – **L**′) ≡ ν_**LL** ^′^_ is shorthand notation, and the subscript **0** is omitted when **L** = **0**.

To approximate ρ^cryst^, an auxiliary density ρ̃^cryst^ is defined as

13 $$\rho^{\mathrm{cryst}}\approx {\mathrm{\rho ̃}}^{\mathrm{cryst}}=\sum_{L}{\mathrm{\rho ̃}}_{L}$$

where each unit cell auxiliary density ρ̃_**L**_ is given by

14 $${\mathrm{\rho ̃}}_{L}=\sum_{\alpha }c^{T}\alpha_{L}$$

Here, α denotes GTOs that form the auxiliary basis functions in **α**. Expansion coefficients **c** are the same across all unit cells and are found by minimizing the Coulomb interaction *D* of the residual density *δρ* = ρ – ρ̃

15 $$D=\iint \delta \rho \left(r\right)\frac{1}{\left|r-r^{\prime }\right|}\sum_{L}\delta \rho_{L}\left(r^{\prime }\right)drdr^{\prime }=\left(\delta \rho |\delta \rho_{L}\right)=\left(\rho -\mathrm{\rho ̃}|\rho_{L}-{\mathrm{\rho ̃}}_{L}\right)$$

In periodic systems, *D* remains finite only if *δρ* is chargeless, requiring

16 $$\int \delta \rho \left(r\right)dr=0\Rightarrow \int \mathrm{\rho ̃}\left(r\right)dr=N_{\mathrm{el}}$$

where *N*_el_ is the total electron count. This constraint allows to decompose ρ̃ into charged (ρ̃_∥_) and chargeless (ρ̃_⊥_) parts

17 $$\mathrm{\rho ̃}={\mathrm{\rho ̃}}_{∥}+{\mathrm{\rho ̃}}_{⊥}=c_{∥}^{T}\alpha +c_{⊥}^{T}\alpha$$

with

18 $$\int {\mathrm{\rho ̃}}_{∥}\left(r\right)dr=N_{\mathrm{el}},\mathrm{and},\int {\mathrm{\rho ̃}}_{⊥}\left(r\right)dr=0$$

Orthogonal projection matrices

19 $$P_{∥}={\mathrm{nn}}^{T},\mathrm{and},P_{⊥}=1-{\mathrm{nn}}^{T}$$

allow the decomposition of **c** as **c**_∥_ = **P**_∥_ **c** and **c**_⊥_ = **P**_⊥_ **c**, where **n** is the normalized auxiliary charge vector with elements *n*_α_ given as

20 $$n_{\alpha }=\frac{1}{\left|q\right|}q_{\alpha },,q={\left(q_{1},q_{2},...\right)}^{T},,q_{\alpha }=\int \alpha \left(r\right)dr$$

Projection of **α** yields the charged (**α**_∥_) and chargeless (**α**_⊥_) auxiliary basis functions: **α**_∥_ = **P**_∥_ **α** and **α**_⊥_ = **P**_⊥_ **α**.

The expansion coefficients of ρ̃_∥_ are

21 $$c_{∥}=\frac{N_{\mathrm{el}}}{\left|q\right|}n$$

and the chargeless part ρ̃_⊥_ is optimized by minimizing *D* with respect to **c**_⊥_ yielding

22 $$\left(V_{⊥}+P_{∥}\right)c_{⊥}=\xi_{⊥}$$

where **V**_⊥_ is the projected Coulomb metric matrix

23 $$V_{⊥}=P_{⊥}{\mathrm{VP}}_{⊥}=\left(\alpha_{⊥}|\alpha_{⊥L}^{T}\right)$$

and **ξ**_⊥_ is given by

24 $$\xi_{⊥}=\left(\alpha_{⊥}|\rho_{L}-{\mathrm{\rho ̃}}_{∥L}\right)$$

This DF scheme guarantees convergent lattice sums in eqs 22–24 by using only chargeless quantities. Unlike other DF approaches for extended systems,^30–32^ this method yields converged lattice sums without adding nuclear charge distributions.

The final auxiliary density coefficients are given by **c** = **c**_∥_ + **c**_⊥_. The Coulomb matrix elements *J*_*μν*_^**L** ^′^^ in real space are calculated as

25 $$J_{\mu \nu }^{L\prime }=\left(\mu \nu_{L\prime }|{\mathrm{\rho ̃}}_{L}-\rho_{nL}\right)$$

where ρ_n**L**_ represents the nuclear charge distribution. Note that the difference ρ̃_**L**_ – ρ_n**L**_ is charge neutral.

The total Coulomb energy is expressed as

26 $$J=\sum_{\mu \nu L^{\prime }}D_{\mu \nu }^{L\prime }J_{\mu \nu }^{L\prime }-\frac{1}{2}\left(\mathrm{\rho ̃}+\rho_{n}|{\mathrm{\rho ̃}}_{L}-\rho_{nL}\right)$$

The DF-accelerated continuous fast multipole method (DF-CFMM) is applied to evaluate Coulomb lattice sums in eqs 22–26 directly in real space.^22–25^ In DF-CFMM the Coulomb lattice sum is divided into a crystal near-field (CNF) and a crystal far-field (CFF) component. For a sum (ρ_1_|ρ_2**L**_), distribution ρ_1_ in the central unit cell interacts with all images ρ_2**L**_ of ρ_2_. The CNF portion accounts for interactions within nearby cells, while the CFF covers distant cells

27 $$\sum_{L}\left(\rho_{1}|\rho_{2L}\right)=\sum_{L\in \mathrm{CNF}}\left(\rho_{1}|\rho_{2L}\right)+\sum_{L\in \mathrm{CFF}}\left(\rho_{1}|\rho_{2L}\right)$$

The CFF sums are computed very efficiently with multipole expansions and recurrence relations, while the CNF part is evaluated in real space using CFMM. Distributions ρ_1_ and ρ_2_ are organized into an octree, decomposing interactions into near-field (NF) and far-field (FF) terms, with CFMM using DF employed to efficiently handle NF interactions.^23^

### Exchange–Correlation Energy and Matrix

The XC energy is obtained as a functional of the density and its derivatives. In the local density approximation (LDA), only the density itself is needed, while the generalized gradient approximation (GGA) necessitates first-order spatial derivatives. For meta-generalized gradient approximations (metaGGAs), the kinetic-energy density τ is further considered. Thus, the XC energy reads

28 $$E_{\mathrm{xc}}=\int f_{\mathrm{xc}}\left[\rho \left(r\right),\gamma \left(r\right),\tau \left(r\right)\right]dr$$

with γ­(**r**) = |∇ρ­(**r**)|^2^ and *f*_xc_ describing the specific density functional approximation. Here, the integration is carried out over the unit cell for periodic systems and over the complete molecule in discrete systems. The XC matrix for the KS equations is formally defined as

29 $$X_{\mu \nu }^{k}=\sum_{L^{\prime }}e^{ik^{T}L\prime }X_{\mu \nu }^{L\prime },\mathrm{with},X_{\mu \nu }^{L\prime }=\int {Ô}_{\mathrm{xc}}\left[\mu \nu_{L\prime }\right]dr$$

where *Ô*_xc_ denotes the XC operator. In practical implementations, the integration is carried out numerically on a grid with

30 $$E_{\mathrm{xc}}=\sum_{i}\sum_{m\in i}w_{m}\times f_{\mathrm{xc}}\left[\rho \left(r_{m}\right),\gamma \left(r_{m}\right),\tau \left(r_{m}\right)\right]$$

31 $$X_{\mu \nu }^{L\prime }=\sum_{i}\sum_{m\in i}X_{\mu \nu }^{L\prime ,m}$$

where *i* are the atoms in the unit cell and *m* is a grid point with its weight *w*_*m*_ leading to

32 $$X_{\mu \nu }^{L\prime ,m}=w_{m}\sum_{L}{Ô}_{\mathrm{xc}}\left[\mu_{L}^{m}\nu_{\mathrm{LL}\prime }^{m}\right]$$

In practice, the XC operator is not used explicitly and its form for metaGGAs is also not easily available.^33^ Instead the matrix elements are obtained from the derivative of the XC energy with respect to the density matrix.

For simplicity, we consider only closed-shell systems in this subsection. Then, the XC matrix reads

33 XμνL′,m=+∑L(μLmzνL+L′,m+zμL,mνLL′)+∑L((∇μL)·tνL+L′,m+tμL,m·(∇νLL′))

with the LDA and GGA potential *z*

34 $$z_{\mu }^{L,m}=\frac{w_{m}}{2}\frac{\partial f_{\mathrm{xc}}}{\partial \rho }\mu_{L}^{m}+2w_{m}\frac{\partial f_{\mathrm{xc}}}{\partial \gamma }\left(\nabla \rho \right)\cdot \left(\nabla \mu_{L}^{m}\right)$$

and the metaGGA potential vector **t**

35 $$t_{\mu ,\alpha }^{L,m}=\frac{w_{m}}{4}\frac{\partial f_{\mathrm{xc}}}{\partial \tau }\left(\nabla_{\alpha }\mu_{L}^{m}\right)$$

where α denotes the Cartesian directions. Here, derivatives of *f*_xc_ are always formed at the respective grid point *m*. The electron density and its derivatives are also evaluated at these grid points according to

36 $$\rho^{m}=\sum_{\mu L}\sum_{\nu L^{\prime }}D_{\mu \nu }^{L\prime -L}\mu_{L}\nu_{L\prime }$$

37 $$\nabla \rho^{m}=\sum_{\mu L}\sum_{\nu L^{\prime }}D_{\mu \nu }^{L\prime -L}\left[\left(\nabla \mu_{L}\right)\nu_{L\prime }+\mu_{L}\left(\nabla \nu_{L\prime }\right)\right]$$

38 $$\tau^{m}=\frac{1}{2}\sum_{\mu L}\sum_{\nu L^{\prime }}D_{\mu \nu }^{L\prime -L}\left(\nabla \mu_{L}\right)\cdot \left(\nabla \nu_{L\prime }\right)$$

For each grid point, only a limited number of basis functions contributes and therefore the computation of the XC matrix scales roughly linearly with the number of grid points.

### Fock Exchange

Fock exchange is an essential ingredient in accurate descriptions of the electronic structure of periodic systems.^34^ Going beyond local and semilocal approximations of XC functionals in DFT, it is required for hybrid and range-separated hybrid-DFT XC functionals, the Hartree–Fock (HF) method, and as a starting point for advanced approaches considering electronic correlations such as the MP2 scheme of Møller–Plesset perturbation theory, the coupled cluster ansatz, or random phase and *GW* approximations.^34–36^

A robust formulation of periodic Fock exchange requires caution because of the artificial periodicity of the density matrix for any finite Born-von Kármán supercell size.^37–39^ From a formal, basis-set-independent point of view the complications for establishing a robust scheme for Fock exchange can be seen in real space as follows.^20^ Considering a spin-degenerate system, the one-electron density matrix is given as

39 $$\rho \left(r_{1},r_{2}\right)=\sum_{p,k}2f_{p}^{k}\psi_{p}^{k}\left(r_{1}\right)\psi_{p}^{k^{*}}\left(r_{2}\right)$$

with the crystal orbital ψ_*p*_^**k**^(**r**_1_) belonging to band *p* and wavevector **k** as well as the occupation number *f*_*p*_^**k**^. Born-von Kármán periodic boundary conditions imply that ψ_*p*_^**k**^(**r**_1_) = ψ_*p*_^**k**^(**r**_1_ + **L**_1_) for any supercell lattice vector **L**_1_. Thus, from the definition of the density matrix ρ­(**r**_1_,**r**_2_), the periodicity ρ­(**r**_1_,**r**_2_) = ρ­(**r**_1_ + **L**_1_,**r**_2_) = ρ­(**r**_1_,**r**_2_ + **L**_1_) in both spatial arguments follows for any finite number of employed *k*-points, but ρ­(**r**_1_,**r**_2_) should decay with distance |**r**_1_ – **r**_2_| in the real crystal.^40–44^ The unphysical periodicity of the off-diagonal elements of the one-electron density matrix ρ­(**r**_1_,**r**_2_) will impact the exchange energy per unit cell of the crystal

40 $$E_{X}=-\frac{1}{4N_{k}}\int_{\Omega }\int \frac{\rho \left(r_{1},r_{2}\right)\rho \left(r_{2},r_{1}\right)}{\left|r_{1}-r_{2}\right|}dr_{1}dr_{2}$$

described by a finite Born-von Kármán supercell. Note that this problem does not appear for the Coulomb energy

41 $$J=\frac{1}{2N_{k}}\int_{\Omega }\int \frac{\rho \left(r_{1},r_{1}\right)\rho \left(r_{2},r_{2}\right)}{\left|r_{1}-r_{2}\right|}dr_{1}dr_{2}$$

which depends only on the diagonal part ρ­(**r**_1_,**r**_1_), i.e., the conventional electron density, which is naturally periodic on every single (primitive) unit cell.

These formal considerations show that exchange matrix elements may be divergent for periodic systems as a result of the artificial periodicity of the off-diagonal elements of the density matrix if no precautions are taken. In ref 20, we have presented a robust implementation of the periodic Fock exchange in TURBOMOLE’s Riper module. We have compared two truncation schemes for a real-space construction, namely the minimum image convention (MIC)^45^ and the truncated Coulomb interaction (TCI).^46^ They both remove the divergence for discrete *k*-meshes by basically restricting off-diagonal elements of the density matrix to just one Born-von Kármán supercell. Calculations with periodic Fock exchange may thus be unstable for small Born-von Kármán supercells, but for a sufficiently large *k* mesh or size of the supercell, stable self-consistent field (SCF) calculations and convergence of total energies are typically achieved. As shown in Table 1, both MIC and TCI regularization schemes converge to the same result, but we find the behavior with the MIC to be generally smoother,^20^ and therefore recommend to use this scheme.

**Table 1:** Self-Consistent HF Total Energies per Primitive Cell in E_h_ for LiH in the Rocksalt Structure with a Lattice Constant of 4.084 Å^46^ ^,^ ^a^

*k*-mesh	MIC	TCI
5 × 5 × 5	–8.06060281	–^b^
7 × 7 × 7	–8.06058890	–^b^
9 × 9 × 9	–8.06058834	–8.06058764
11 × 11 × 11	–8.06058830	–8.06058831
13 × 13 × 13	–8.06058829	–8.06058829
19 × 19 × 19	–8.06058829	–8.06058829

a Primitive unit cells with two atoms are calculated for different *k*-meshes using the pob-TZVP basis set.^47^ Adapted with permission from ref 20. Copyright 2018 American Chemical Society.

b Born-von Kármán supercell too small.

The size of the Born-von Kármán supercell or *k*-mesh that is required for a reliable exchange energy depends on the locality of the density matrix and hence both on the electronic structure of the studied material and on the chosen basis set. For selected insulators and semiconductors, we have demonstrated that the HF total energy converges exponentially with the number of *k*-points.^20^

Through our implementation of periodic exchange,^20^ conventional HF calculations can be carried out for periodic systems of any dimension. In addition, DFT calculations with global and range-separated hybrid functionals can now be performed routinely for semiconductors and insulators, and we showed successful application^20^ of PBE0 ^48^ and HSE06 ^49^ functionals, respectively. As the next important step, analytical gradients are needed for structure optimization. The existing Fock exchange infrastructure can now be used for applying advanced electronic structure methods to periodic systems that require an exact exchange.

## Extension to a Relativistic Framework

In heavy elements, the electrons in the vicinity of the nucleus move at a speed close to that of light. Therefore, not only the laws of quantum mechanics but also those of special relativity need to be considered. This leads to the framework of relativistic quantum mechanics.^50,51^ Here, special relativity can be described with either pseudopotentials and effective core potentials^52^ (ECPs) or all-electron approaches based on the Dirac equation.^53–56^ In terms of computational costs, ECPs are beneficial, as they ”cut out” the core electrons and introduce a pseudopotential, which accounts for relativistic effects. The corresponding pseudopotential is parametrized based on all-electron calculations. ECPs are sufficient for properties that are associated with the valence electrons such as the chemical bonding or the structure. Therefore, we will consider relativistic effects with ECPs in this work.

Relativistic effects are generally partitioned into scalar or spin-independent contributions and spin-dependent effects, such as spin–orbit interaction. Scalar-relativistic effects describe the mass-velocity relation and the Darwin term. These effects do not lead to structural changes of the KS equations, i.e., the scalar ECP is included in the equations just like the electron–nucleus Coulomb potential. Therefore, the standard one-component (1c) formalism with separate α and β spin space is sufficient. However, spin–orbit coupling requires further generalizations, as the spin is not a good quantum number anymore. That is, the KS wave functions are no longer eigenfunctions of the spin operator. In other words, the wave function is a combination of α and β spin contributions. Moreover, the spin–orbit ECPs are described with complex operators in position space. These considerations lead to the two-component (2c) Hamiltonian in the Born–Oppenheimer approximation given by

42 $$Ĥ=\mathrm{T̂}+Ĵ+\left[\sigma_{0}{\mathrm{V̂}}^{0}+\sigma ◦{\mathrm{V̂}}^{\mathrm{SO}}\right]+{\mathrm{V̂}}_{\mathrm{NN}}$$

Here, *V̂* ^0^ denotes the scalar-relativistic ECPs or pseudpotentials, whereas **V̂** ^SO^ refers to the spin–orbit ECPs. Note that **V̂** ^SO^ is a vector consisting of three spin components. σ_0_ and **σ** are the (2 × 2) identity matrix and the vector of the three Pauli spin matrices, respectively. *T̂*, *Ĵ*, and *V̂*_NN_ are the kinetic-energy operator, the Coulomb interaction, and the nucleus–nucleus potential. The operators are of the same form as in nonrelativistic quantum chemistry. For clarity, we use · to indicate a scalar product associated with spatial indices, ◦ for the scalar product of the spin components, and ⊙ for the simultaneous scalar product. Bold letters are used for vectors with spin and spatial components, as well as matrix representations of operators.

The KS Bloch functions are now linear combinations of the α and β spin contributions. That is, they are so-called spinors within the LCAO ansatz defined as

43 $$\psi_{p}^{k}\left(r\right)=\left(\begin{array}{l} \psi_{p\alpha }^{k}\left(r\right)\\ \psi_{p\beta }^{k}\left(r\right) \end{array}\right)=\sum_{\mu }\left(\begin{array}{l} C_{\mu p\alpha }^{k}\\ C_{\mu p\beta }^{k} \end{array}\right)\phi_{\mu }^{k}\left(r\right)$$

where ϕ_μ_^**k**^ is a one-component and spin-independent Bloch atomic orbital

44 $$\phi_{\mu }^{k}\left(r\right)=\frac{1}{\sqrt{N_{\mathrm{UC}}}}\sum_{L}e^{ik^{T}L}\mu_{L}\left(r\right)$$

Therefore, the expansion coefficients *C*_*μp*_ are always complex—even for molecules and at the Γ point. However, the atomic orbitals μ_**L**_(**r**) are still real and the same as in a nonrelativistic calculation. The two-component KS equations in this representation follow as

45 $$\left(\begin{array}{ll} F_{\alpha \alpha }^{k} & F_{\alpha \beta }^{k}\\ F_{\beta \alpha }^{k} & F_{\beta \beta }^{k} \end{array}\right)\left(\begin{array}{l} C_{\alpha }^{k}\\ C_{\beta }^{k} \end{array}\right)=\left(\begin{array}{ll} S^{k} & 0\\ 0 & S^{k} \end{array}\right)\left(\begin{array}{l} C_{\alpha }^{k}\\ C_{\beta }^{k} \end{array}\right)\epsilon^{k}$$

In real space, the elements of the KS matrix read

46 $$F_{\alpha \alpha }^{L}=T^{L}+J^{L}+V^{0,L}+V_{z}^{\mathrm{SO},L}+X_{\alpha \alpha }^{L}+K_{\alpha \alpha }^{L}$$

47 $$F_{\alpha \beta }^{L}=V_{x}^{\mathrm{SO}L}-iV_{y}^{\mathrm{SO},L}+X_{\alpha \beta }^{L}+K_{\alpha \beta }^{L}$$

48 $$F_{\beta \beta }^{L}=T^{L}+J^{L}+V^{0,L}-V_{z}^{\mathrm{SO},L}+X_{\beta \beta }^{L}+K_{\beta \beta }^{L}$$

This clearly shows that the α and β spins are coupled. The kinetic-energy and Coulomb terms are identical to the nonrelativistic limit.^22,23,27^ The KS coefficients **C** ^**k**^ can be used to construct a 2c density matrix in reciprocal space in the same way as that done in the 1c approach. To exploit sparsity, the integral evaluation for the KS matrix is also done in the position space. Here, the 2c density matrix from the Fourier transformation reads

49 $$D^{L}=\left(\begin{array}{ll} D_{\alpha \alpha }^{L} & D_{\alpha \beta }^{L}\\ D_{\beta \alpha }^{L} & D_{\beta \beta }^{L} \end{array}\right),\mathrm{with},{\left(D^{L}\right)}^{†}=D^{-L}$$

To extend an existing 1c code to a 2c framework with minimal effort, linear combinations of the spin blocks are formed.^57,58^ This not only reduces memory requirements as the information encoded in the 2c density matrix is redundant but also allows one to reuse large parts of the integral and transformation code. For periodic systems, we form the real symmetric (RS), real antisymmetric (RA), imaginary antisymmetric (IA), and imaginary symmetric (IS) linear combinations as^59^

50 [Dσσ′RS,RA]L=12[Re(Dσσ′±Dσ′σ)]L

51 [Dσσ′IA,IS]L=12[Im(Dσσ′±Dσ′σ)]L

Note that the same-spin antisymmetric contributions are zero and symmetry or antisymmetry of the matrix **M** for periodic systems refers to the relation

52 $$M_{\mu \nu }^{L}=\pm M_{\nu \mu }^{-L}$$

To compare with, only real and symmetric parts are necessary to describe the matrices in position space within a scalar-relativistic formalism.

The exact exchange term can be easily computed based on an existing 1c implementation with minor modifications to include the antisymmetric linear combinations.^59^ The full exact exchange matrix is constructed by reversing the linear combinations above. Furthermore, these linear combinations can be used to describe all physical quantities, such as the electron density and current density. The symmetric contributions constitute the particle density ρ and the spin magnetization vector **m** according to

53 $$\rho^{L}=\sum_{\mu \nu L^{\prime }}{\left[D_{\alpha \alpha }^{\mathrm{RS}}+D_{\beta \beta }^{\mathrm{RS}}\right]}_{\mu \nu }^{L\prime }\mu_{L}\nu_{\mathrm{LL}\prime }$$

54 $$m_{x}^{L}=\sum_{\mu \nu L^{\prime }}2{\left[D_{\alpha \beta }^{\mathrm{RS}}\right]}_{\mu \nu }^{L\prime }\mu_{L}\nu_{\mathrm{LL}\prime }$$

55 $$m_{y}^{L}=\sum_{\mu \nu L^{\prime }}2{\left[D_{\alpha \beta }^{\mathrm{IS}}\right]}_{\mu \nu }^{L\prime }\mu_{L}\nu_{\mathrm{LL}\prime }$$

56 $$m_{z}^{L}=\sum_{\mu \nu L^{\prime }}{\left[D_{\alpha \alpha }^{\mathrm{RS}}-D_{\beta \beta }^{\mathrm{RS}}\right]}_{\mu \nu }^{L\prime }\mu_{L}\nu_{\mathrm{LL}\prime }$$

so that the total 2c spin density^60^ ρ_s_^**L**^ = 1/2 (ρ^**L**^σ_0_ + **m** ^**L**^◦**σ**) follows as

57 $$\rho_{s}^{L}=\frac{1}{2}\left(\begin{array}{ll} \rho^{L}+m_{z}^{L} & m_{x}^{L}-im_{y}^{L}\\ m_{x}^{L}+im_{y}^{L} & \rho^{L}-m_{z}^{L} \end{array}\right)$$

Note that summation over the cell densities again yields the crystal densities. Compared to the scalar unrestricted Kohn–Sham (UKS) formalism, all spin directions are simultaneously considered, and the norm of **m** results in the spin expectation value. Other density variables such as the kinetic-energy density τ are obtained in the same manner, i.e., only the basis function term on the right-hand side, μ_**L**_ ν_**LL**′_, is replaced with 1/2­[***∇*** μ_**L**_]­[***∇*** ν_**LL**′_]. The antisymmetric linear combinations give rise to the particle current density **j** and the spin current densities **Y**_*u*_ with *u* ∈ {*x*,*y*,*z*} referring to the spin components.

58 $$j^{L}=-\frac{1}{2}\sum_{\mu \nu L^{\prime }}{\left[D_{\alpha \alpha }^{\mathrm{IA}}+D_{\beta \beta }^{\mathrm{IA}}\right]}_{\mu \nu }^{L\prime }\eta_{\mu \nu }$$

59 $$Y_{x}^{L}=-\frac{1}{2}\sum_{\mu \nu L^{\prime }}2{\left[D_{\alpha \beta }^{\mathrm{IA}}\right]}_{\mu \nu }^{L\prime }\eta_{\mu \nu }$$

60 $$Y_{y}^{L}=-\frac{1}{2}\sum_{\mu \nu L^{\prime }}2{\left[D_{\alpha \beta }^{\mathrm{RA}}\right]}_{\mu \nu }^{L\prime }\eta_{\mu \nu }$$

61 $$Y_{z}^{L}=-\frac{1}{2}\sum_{\mu \nu L^{\prime }}{\left[D_{\alpha \alpha }^{\mathrm{IA}}-D_{\beta \beta }^{\mathrm{IA}}\right]}_{\mu \nu }^{L\prime }\eta_{\mu \nu }$$

62 $$\eta_{\mu \nu }=\left[\nabla \mu_{L}\right]\nu_{\mathrm{LL}\prime }-\mu_{L}\left[\nabla \nu_{\mathrm{LL}\prime }\right]$$

These current densities are only due to the spin–orbit potential, i.e., no current densities arise in a field-free nonrelativistic or scalar-relativistic 1c ground-state calculation. That is, spin–orbit coupling is a form of magnetic induction.^50,61^ This also means that the kinetic-energy density necessitates a generalization to ensure gauge invariance and the von-Weizsäcker inequality for the iso-orbital condition.^16,17,62–68^ In other words, the functional should depend on the density, its gradient, the kinetic-energy density, and the current density.

In the following, we will first consider a closed-shell system for simplicity.^15–17,67^ For a closed-shell Kramers-restricted system, the spin magnetization and particle current density vanish. However, the three spin current densities are still generally nonzero. The generalized kinetic-energy density for metaGGAs is then defined as^16,17,67^

63 $$\mathrm{\tau ̃}=\tau -\frac{Y⊙Y}{2\rho }$$

where τ is the standard kinetic-energy density. Note that the current densities are not included for LDAs and GGAs. This directly introduces a dependence of the semilocal XC functional term on the current density. Therefore, the XC energy follows as

64 $$E_{\mathrm{xc}}=\int f_{\mathrm{xc}}\left[n\left(r\right),\gamma \left(r\right),\tau \left(r\right),Y\left(r\right)\right]dr=\int g_{\mathrm{xc}}\left[n\left(r\right),\gamma \left(r\right),\mathrm{\tau ̃}\left(r\right)\right]dr$$

Following the numerical schemes as done in the nonrelativistic limit, this leads to the 2c XC matrix according to^16,17,67^

65 $$X_{\mu \nu }^{L\prime ,m}=\sigma_{0}\sum_{L}\left(\mu_{L}^{m}z_{\nu }^{L+L\prime ,m}+z_{\mu }^{L,m}\nu_{\mathrm{LL}\prime }\right)+\sigma_{0}\sum_{L}\left(\left(\nabla \mu_{L}^{m}\right)\cdot t_{\nu }^{L+L\prime ,m}+t_{\mu }^{L,m}\cdot \left(\nabla \nu_{\mathrm{LL}\prime }\right)\right)+i\sigma ◦\sum_{L}\left(y_{\mu }^{L,m}\nu_{\mathrm{LL}\prime }-\mu_{L}y_{\nu }^{L+L\prime ,m}\right)$$

with the modified LDA and GGA potential *z*

66 $$z_{\mu }^{L,m}=\frac{w_{m}}{2}\frac{\partial g_{\mathrm{xc}}}{\partial \rho }\mu_{L}^{m}+\frac{w_{m}}{2}\frac{Y_{m}⊙Y_{m}}{2\rho_{m}^{2}}\frac{\partial g_{\mathrm{xc}}}{\partial n}\mu_{L}^{m}+2w_{m}\frac{\partial g_{\mathrm{xc}}}{\partial \gamma }\left(\nabla \rho_{m}\right)\cdot \left(\nabla \mu_{L}^{m}\right)$$

and the metaGGA potential vector **t** in real space

67 $$t_{\mu ,\alpha }^{L,m}=\frac{w_{m}}{4}\frac{\partial g_{\mathrm{xc}}}{\partial \tau }\left(\nabla_{\alpha }\mu_{L}^{m}\right)$$

Note that the GGA and metaGGA contributions are the same as in the nonrelativistic limit. However, the spin current densities lead to a new term in the LDA part of *z*. The spin current densities further lead to the vector **y** in spin space

68 $${\left(y_{\mu }^{L,m}\right)}_{u}=\frac{w_{m}}{2}\frac{\partial g_{\mathrm{xc}}}{\partial \mathrm{\tau ̃}}\frac{{\left(Y_{m}\right)}_{u}}{\rho_{m}}\cdot \left(\nabla \mu_{L}^{m}\right)$$

which arises from the application of the chain rule, see ref 67 for details.

For open-shell systems, time-reversal symmetry does not hold for a single-reference KS solution.^69^ Here, two distinct formalisms are available in TURBOMOLE, namely the canonical^15,57,60,67^ and the Sclamani–Frisch^15,17,59,70–72^ approach. The former is more common and utilizes a projection onto spin magnetization. The Scalmani–Frisch approach for current-dependent metaGGAs is presented in ref 17 and we note that this formalism avoids the projection onto the spin magnetization vector **m**, which is crucial to naturally ensure the closed-shell limit with all three spin current densities contributing. Note that validation is strictly possible for the molecular limit by comparison to calculations in finite magnetic fields. Here, the current density is required for gauge-origin independence and translational invariance.^17,33,66,67^

Not only spin–orbit coupling induces a current density but magnetic properties^73–76^ or electromagnetic perturbations in general.^77^ Thus, the 2c implementation could be easily modified to use explicitly current-dependent metaGGAs in 1c RT-TDDFT calculations, as described in the Supporting Information. Here, the IA contribution of the propagated density matrix is used to compute the current density. The spin channels are still fully decoupled in the scalar-relativistic or nonrelativistic RT-TDDFT framework. That is, the 1c UKS RT-TDDFT code requires only the particle current density and the spin-z current density (or the α + β and α – β current densities). Likewise, the restricted KS code only necessitates the particle current density. As shown in the Supporting Information, comparison of RT-TDDFT and the established linear-response TDDFT framework^77,78^ with the current density contribution to the kernel further validated our XC potential routines for current-dependent metaGGAs.

## DFT-Based Embedding

DFT-based embedding methods offer a practical approach to modeling complex chemical systems by partitioning the total system into a smaller active subsystem, which is the region of interest, and a larger environment subsystem. This allows for the application of complex or higher-level methods to the active subsystem while treating the less critical environment at the DFT level. Thus, this allows the study of large systems efficiently.

Frozen density embedding (FDE)^79^ and projection-based embedding (PbE)^80^ are two prominent DFT-based embedding techniques. FDE relies on a frozen environment density and an approximate kinetic-energy density functional (KEDF) to account for the interaction among the subsystems. PbE, on the other hand, employs a level-shift projection operator to enforce orthogonality between the subsystem orbitals, eliminating the need for approximate KEDFs and enabling exact embedding calculations, which are especially useful for strongly interacting subsystems.

This section gives a concise overview of FDE and PbE, as well as the relevant expressions for the embedding potentials.

### Frozen-Density Embedding

FDE, introduced by Wesołowski and Warshel,^79^ is based on the concept of a frozen environment density. The total electronic density (ρ^tot^) of the system is expressed as the sum of the individual active and environment subsystem densities, ρ^act^ and ρ^env^, respectively

69 $$\rho^{\mathrm{tot}}\left(r\right)=\rho^{\mathrm{act}}\left(r\right)+\rho^{\mathrm{env}}\left(r\right)$$

The total energy (*E* ^tot^) of the system can be written as a bifunctional of the subsystem densities

70 $$E^{\mathrm{tot}}\left[\rho^{\mathrm{act}},\rho^{\mathrm{env}}\right]=E^{\mathrm{act}}\left[\rho^{\mathrm{act}}\right]+E^{\mathrm{env}}\left[\rho^{\mathrm{env}}\right]+E^{\mathrm{int}}\left[\rho^{\mathrm{act}}\rho^{\mathrm{env}}\right]$$

Here, *E* ^act^ and *E* ^env^ are the individual subsystem energies calculated using standard KS-DFT, and *E* ^int^ represents the interaction energy between the subsystems

71 $$E^{\mathrm{int}}\left[\rho^{\mathrm{act}},\rho^{\mathrm{env}}\right]=\int \rho^{\mathrm{act}}\left(r\right)v_{\mathrm{nuc}}^{\mathrm{env}}\left(r\right)dr+\int \rho^{\mathrm{env}}\left(r\right)v_{\mathrm{nuc}}^{\mathrm{act}}\left(r\right)dr+E_{\mathrm{nuc}}^{\mathrm{act},\mathrm{env}}+\iint \frac{\rho^{\mathrm{act}}\left(r\right)\rho^{\mathrm{env}}\left(r^{\prime }\right)}{\left|r-r^{\prime }\right|}drdr^{\prime }+E_{\mathrm{xc}}^{\mathrm{nadd}}\left[\rho^{\mathrm{act}},\rho^{\mathrm{env}}\right]+T_{s}^{\mathrm{nadd}}\left[\rho^{\mathrm{act}},\rho^{\mathrm{env}}\right]$$

*E* ^int^ includes contributions from the electrostatic interaction between the nuclei of the two subsystems (∫ρ^act^(**r**)*v*_nuc_^env^ **r**)*d* **r** + ∫ρ^env^(**r**)*v*_nuc_^act^(**r**)*d* **r**); the Coulomb repulsion between the electrons of the active and environment subsystems $\left(\iint \frac{\rho^{\mathrm{act}}\left(r\right)\rho^{\mathrm{env}}\left(r^{\prime }\right)}{\left|r-r^{\prime }\right|}drdr^{\prime }\right)$ ; nonadditive contributions to the kinetic-energy (*T*_*s*_^nadd^) and XC energy (*E*_xc_^nadd^) arising from the interaction between the subsystems. The nonadditive terms account for the nonlinear nature of the kinetic and XC functionals. Since the exact form of *T*_*s*_^nadd^ is unknown, approximations have to be employed in practical applications. Therefore, the accuracy of FDE is limited for systems with strongly overlapping densities, as the approximate KEDFs may not accurately account for Pauli repulsion between subsystem electrons.

#### Embedding Potential

The density of the active subsystem (ρ^act^) in the presence of a given frozen environment density (ρ^env^) is obtained by minimizing the total energy bifunctional (eq 70) with respect to ρ^act^ while keeping ρ^env^ fixed. This minimization leads to a set of KS-like equations known as the Kohn–Sham constrained electron density (KSCED) equations

72 $$\left[-\frac{\nabla^{2}}{2}+v_{\mathrm{eff}}^{\mathrm{KSCED}}\left(\rho^{\mathrm{act}},\rho^{\mathrm{env}},v_{\mathrm{nuc}}^{\mathrm{env}}\right)\right]\phi_{i}^{\mathrm{act}}\left(r\right)=\epsilon_{i}\phi_{i}^{\mathrm{act}}\left(r\right)$$

with *i* = 1, ..., *N* ^act^.

The effective KSCED potential (*v*_eff_^KSCED^) consists of the standard KS potential (*v*_eff_^KS^) of the active subsystem and the embedding potential (*v*_emb_), that accounts for the interaction between the active and environment subsystems and is given by

73 $$v_{\mathrm{emb}}\left[\rho^{\mathrm{act}},\rho^{\mathrm{env}},v_{\mathrm{nuc}}^{\mathrm{env}}\right]=v_{\mathrm{nuc}}^{\mathrm{env}}\left(r\right)+\int \frac{\rho^{\mathrm{env}}\left(r^{\prime }\right)}{\left|r-r^{\prime }\right|}dr^{\prime }+\frac{\delta E_{\mathrm{xc}}^{\mathrm{nadd}}\left[\rho^{\mathrm{act}},\rho^{\mathrm{env}}\right]}{\delta \rho^{\mathrm{act}}\left(r\right)}+\frac{\delta T_{s}^{\mathrm{nadd}}\left[\rho^{\mathrm{act}},\rho^{\mathrm{env}}\right]}{\delta \rho^{\mathrm{act}}\left(r\right)}$$

The embedding potential includes contributions from the nuclear potential of the environment (*v*_nuc_^env^(**r**)), the Coulomb potential due to the electrons of the environment ( $\int \frac{\rho^{\mathrm{env}}\left(r^{\prime }\right)}{\left|r-r^{\prime }\right|}dr^{\prime }),$ the nonadditive XC potential ( $\frac{\delta E_{\mathrm{xc}}^{\mathrm{nadd}}\left[\rho^{\mathrm{act}},\rho^{\mathrm{env}}\right]}{\delta \rho^{\mathrm{act}}\left(r\right)}$ ), and nonadditive kinetic potential ( $\frac{\delta T_{s}^{\mathrm{nadd}}\left[\rho^{\mathrm{act}},\rho^{\mathrm{env}}\right]}{\delta \rho^{\mathrm{act}}\left(r\right)}$ ) terms.

While originally formulated for DFT-in-DFT embedding, the embedding potential described above has been extensively utilized for a variety of embedding calculations. These include applications where the active subsystem is treated using correlated wave function theory (WFT) methods (WFT-in-DFT),^81,82^ many-body perturbation theory methods such as *GW* approximation combined with the Bethe–Salpeter equation (*GW*/BSE-in-DFT),^83–85^ and RT-TDDFT-in-DFT.^21,86,87^

While the exact KEDF remains elusive, it would yield a *v*_emb_ that, within the FDE framework, could accurately reproduce the total KS density (ρ^tot^), provided that the environment density (ρ^env^) is non-negative and the active subsystem density (ρ^act^) is *v*_*s*_-representable.^88^ This implies that ρ^env^ should never be greater than ρ^tot^ at any point in space, thus ensuring that ρ^act^ = ρ^tot^ – ρ^env^ remains non-negative everywhere. However, in practical applications, fulfilling this criterion is often challenging. Numerous choices for ρ^env^ can lead to negative regions in ρ^act^, thereby restricting the choice of usable frozen densities.^89^

#### Freeze-and-Thaw and Subsystem DFT

To address the limitations of the fixed environment density assumption in FDE, the freeze-and-thaw (FaT) procedure was introduced.^90^ In FaT, the roles of the active and environment subsystems are iteratively switched, allowing for self-consistent determination of subsystem densities. This iterative process, in principle, leads to more accurate and mutually polarized subsystem densities.

Once the FaT procedure is performed, there is no formal distinction between the active and environment subsystems, leading to the formulation of subsystem DFT (sDFT).

For a more detailed review on FDE and sDFT, we refer the reader to the reviews in refs 91,92 .

### Projection-Based Embedding

PbE is an exact DFT-based embedding method, that eliminates the need for a nonadditive kinetic potential in *v*_emb_, thereby avoiding the use of approximate KEDFs.^80^ This is done by enforcing orthogonality between the subsystem orbitals using a projection operator, allowing the total kinetic energy to be expressed as the sum of the individual subsystem kinetic energies. This makes PbE suitable for even strongly overlapping subsystem densities.^21,93,94^

Various projection operators have been suggested in the literature such as the level-shift projection operator (LSPO),^80^ Huzinaga operator^95^ and Hoffman operator.^96^ The LSPO has been implemented in TURBOMOLE, defined as

74 $$O_{P}=\lim_{\mu \to \infty }\mu P^{\mathrm{env}}=\lim_{\mu \to \infty }\mu S^{\mathrm{act},\mathrm{env}}D^{\mathrm{env}}S^{\mathrm{env},\mathrm{act}}$$

where μ is the scaling factor (ideally ∞ but in practical implementations, set to 10^6^ a.u.), **D** ^env^ is the density matrix of the environment, and **S** ^act,env^/**S** ^env,act^ are the overlap matrices between the basis functions of the two subsystems.

The LSPO replaces the nonadditive kinetic potential, when *v*_emb_ is written in the matrix form **V**_emb_

75 $$V_{\mathrm{emb}}=V_{\mathrm{nuc}}^{\mathrm{env}}+J_{\mathrm{elec}}^{\mathrm{env}}+X_{\mathrm{nadd}}+O_{P}$$

where **V**_nuc_^env^ and **J**_elec_^env^ are the environmental nuclear and Coulomb potential matrices, respectively, and **X**_nadd_ is the nonadditive XC potential matrix.

The approach developed by Manby et al. relies on prior knowledge of the KS orbitals of the entire system, which are subsequently partitioned into active and environmental subsystems.^80^ In contrast, Chulhai and Jensen proposed a more efficient method that begins with arbitrary subsystem KS orbitals and uses FaT cycles to converge to the exact subsystem densities.^93^ The latter approach is implemented in TURBOMOLE.^21^

## RT-TDDFT and Its Extension to DFT-Based Embedding

RT-TDDFT is a robust method to study the time-dependent behavior of electrons under external electric fields. It achieves this by propagating the KS wave function in time, governed by an effective potential derived from the time-dependent electron density, ρ­(**r**,*t*). For practical purposes, particularly when Gaussian basis sets are employed, the evolution of the reduced single-particle density matrix is preferred. This is expressed through the Liouville–von Neumann (LvN) equation

76 $$i\frac{\partial D^{\prime }\left(t\right)}{\partial t}=F_{p}^{\prime }\left(t\right)D^{\prime }\left(t\right)-D^{\prime }\left(t\right)F_{p}^{\prime }\left(t\right)$$

where **D**′(*t*) and **F**_*p*_^′^(*t*) are the time-dependent density matrix and the perturbed KS matrix, respectively, in the orthonormal molecular orbital basis. Within the dipole approximation, the perturbed KS matrix in the atomic orbital basis is expressed as

77 $$F_{p}\left(t\right)=F\left(t\right)+F^{E}\left(t\right)$$

where **F**(*t*) represents the KS matrix in the absence of external fields, and its implicit time-dependence is induced by **D**(*t*). The term **F** ^E^(*t*) represents the field-induced contributions, given by

78 $$F_{\mathrm{ij}}^{E}=-\sum_{k=x,y,z}M_{\mathrm{ij}}^{k}E_{k}$$

where *M*_*ij*_^*k*^ are the elements of the dipole moment matrices **M** ^*k*^, defined as

79 $$M_{\mathrm{ij}}^{k}=-\int \chi_{i}\left(r\right)k\chi_{j}\left(r\right)dr$$

To propagate the system in time, the LvN equation is integrated using numerical methods.^97^ The implementation in TURBOMOLE employs the Magnus expansion to ensure a unitary propagator, which preserves the idempotency of **D**′.^98,99^ Details of the implementation and its application to linear-response absorption spectra can be found in ref 18. Recently, this implementation was adapted to the strong-field regime to enable simulations of ultrafast phenomena, including high harmonic generation (HHG), under intense laser pulses.^19^ The extension to current-dependent metaGGAs and hybrids is briefly discussed in the Supporting Information of the present work.

The coupling of RT-TDDFT with DFT-based embedding has been a topic of significant interest recently.^86,87^ This coupling, referred to as real-time time-dependent density functional embedding theory (RT-TDDFET) in this work, allows evolving the active subsystem’s electron density while keeping the environment density fixed. RT-TDDFET with LSPO- or KEDF-based embedding potentials is straightforward to implement. The density matrix of the active subsystem, **D**′^act^(*t*), evolves according to

80 $$i\frac{\partial D^{\prime \mathrm{act}}\left(t\right)}{\partial t}=F_{p\;\mathrm{emb}}^{\prime \mathrm{act}}\left(t\right)D^{\prime \mathrm{act}}\left(t\right)-D^{\prime \mathrm{act}}\left(t\right)F_{p\;\mathrm{emb}}^{\prime \mathrm{act}}\left(t\right)$$

where **F**_*p* emb_^′act^(*t*) is the embedded KS matrix of the active subsystem, in the presence of an external field

81 $$F_{p\;\mathrm{emb}}^{\mathrm{act}}\left(t\right)=F^{\mathrm{act}}\left(t\right)+V_{\mathrm{emb}}\left(t\right)+F^{E}\left(t\right)$$

with **V**_emb_(*t*) representing the embedding potential matrix. Depending on the embedding approach, **V**_emb_(*t*) can be KEDF-based (as in FDE) or LSPO-based (as in PbE).

RT-TDDFET framework enables the study of ultrafast phenomena and spectroscopic properties in complex and hybrid chemical systems.

## DFT-Based Embedding Coupled with WFT (WFT-in-DFT)

Correlated WFT methods address limitations of KS-DFT with standard LDA/GGA functionals, such as poor treatment of van der Waals interactions, charge transfer, and strongly correlated systems by providing a systematic approach to include electron correlation. However, state-of-the-art methods like coupled cluster singles and doubles with perturbative triples CCSD­(T), the “gold standard” for ground-state quantum chemistry, scale as *O*(*N* ^7^), making them feasible only for small systems with fewer than 50 atoms. The combination of DFT-based embedding with correlated WFT methods, referred to as WFT-in-DFT, has proven to be an effective way to improve the accuracy of both ground and excited state properties beyond standard DFT^81,82,91,94,100–102^ for complex and large chemical systems. This method has been applied in various contexts, including both KEDF- and LSPO-based embedding. The key advantage of WFT-in-DFT is that it allows for the computationally expensive correlated WFT calculations to be confined only to the region of interest, or the active subsystem, while treating the surrounding environment with DFT. This reduces the overall computational cost significantly while still providing highly accurate results for the active region.

WFT-in-DFT has been particularly successful in accelerating studies of solvated molecules through molecule-in-molecule embedding^101,103^ and in the study of adsorbed molecules on periodic slabs via molecule-in-periodic embedding.^94,102^ In WFT-in-DFT, the embedding potential matrix (**V**_emb_) is added to the HF Hamiltonian of the active subsystem to obtain the HF orbitals. A correlated post-HF method, such as CCSD, is then applied to improve the description of the active subsystem’s ground and excited states. Ideally, **V**_emb_, which depends on the active subsystem’s density matrix, should be updated during both the HF and WFT calculations. However, in this work, **V**_emb_ remains fixed and is constructed from DFT level subsystem density matrices. The total system’s ground-state energy (*E*_tot_^WFT‑in‑DFT^) is calculated by adding a correction term to the DFT total energy (*E*_tot_^DFT^), corresponding to the active region

82 $$E_{\mathrm{tot}}^{\mathrm{WFT}‐\mathrm{in}‐\mathrm{DFT}}=E_{\mathrm{tot}}^{\mathrm{DFT}}+\left(E_{\mathrm{act}}^{\mathrm{WFT}}-E_{\mathrm{act}}^{\mathrm{DFT}}\right)$$

Thus, WFT-in-DFT provides a computationally efficient way to account for the local correlation in the active region.

## DFT-Based Embedding Coupled with Many-Body Perturbation Theory and Bethe–Salpeter Equation (GW/BSE-in-DFT)

Green’s function-based many-body perturbation theory methods, such as the *GW* approximation^104–106^ combined with the Bethe–Salpeter equation^107^ (*GW*/BSE), provide highly accurate excitation energies and exciton binding energies. The *GW* approximation improves upon DFT by considering dynamic screening of the Coulomb interaction, yielding more accurate quasiparticle energies. The BSE then incorporates electron–hole interactions, which are crucial for optical excitations. Combining these (*GW*/BSE) provides accurate optical properties.^108^ However, the high computational cost of full *GW*/BSE calculations limits their applicability to large systems (*O*(*N* ^4^) scaling with the system size).

Therefore, combining *GW*/BSE with DFT-based embedding offers a powerful approach to extend its applicability to larger and more complex systems.^83–85,109^ This embedded approach, termed *GW*/BSE-in-DFT, is similar to the WFT-in-DFT discussed above and allows for the treatment of the active subsystem using *GW*/BSE, while describing the surrounding environment using DFT. This significantly reduces the overall computational cost.

*GW*/BSE-in-DFT calculations are carried out by using the embedded KS orbitals of the active subsystem to calculate the *GW*-in-DFT quasiparticle energies, which are then used to determine the BSE for excitation energy evaluation. For more details regarding the implementation, the readers are referred to ref 109.

## Web-Based Interface for Creating Input Files and Analyzing Output Files

One of the primary hurdles for new users of any quantum chemistry package is the generation of correct input files, which requires a deep understanding of various keywords and calculation setup procedures. This is where a user-friendly, web-based (hence cross-platform) graphical user interface (GUI) application becomes crucial, significantly reducing the learning curve and making the software more accessible to a wider range of researchers.

To this end, Riper-Tools,^110^ a web-based GUI application has been developed to simplify the use of the Riper module in TURBOMOLE. Built using the Streamlit framework,^111^ Riper-Tools leverages several powerful Python libraries under the hood to provide a comprehensive suite of tools for creating input files involving molecular and periodic structures, parsing and visualizing output files, as well as modeling capabilities. Figure 1 shows screenshots of some of the functionalities of the Riper-Tools.

**Figure 1:**
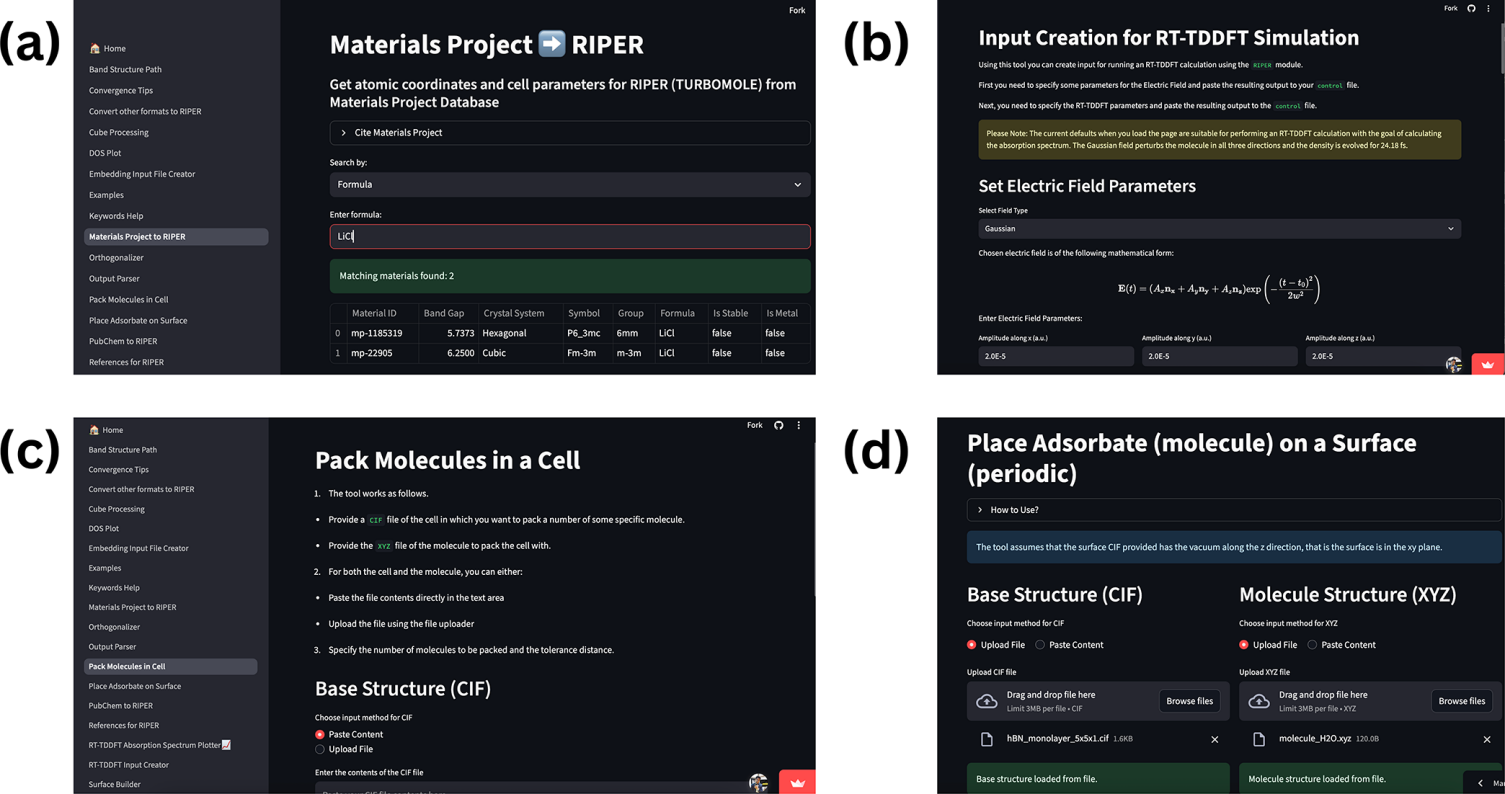
Screenshots of Riper-Tools Web App functionalities: (a) Retrieving crystal structures from the Materials Project, (b) Generating RT-TDDFT input files, (c) Packing molecules into a periodic cell, and (d) Placing adsorbates (molecules) on surfaces.

The key components powering Riper-Tools include:

- **Streamlit**: An open-source Python library that supports the development of interactive web applications with minimal coding effort. Its popularity in the machine learning community is now expanding to computational chemistry, where it enables the development of accessible and interactive scientific applications.^111^
- **Py3Dmol**: This library provides in-browser, interactive 3D visualization of chemical structures, enhancing user experience.^112,113^

- **Atomic Simulation Environment (ASE)**: ASE offers a standardized interface for handling atomic structures and performing operations such as creating supercells, structural translations, and adsorbate placements.^114^ These features facilitate the preparation and manipulation of structures required for periodic simulations.
- **Python Materials Genomics (Pymatgen)**: With its powerful capabilities for handling periodic structures, Pymatgen^115^ supports essential tasks such as file format conversions, symmetry analysis,^116^ and access to materials databases like the Materials Project,^117^ thus enriching the tool’s versatility for periodic system modeling.

### Notable Features of Riper-Tools

Riper-Tools offers a rich set of features designed to streamline various calculations using Riper, including:

- Input Generation
  - Importing structures from popular databases like Materials Project^117^ and PubChem,^118^ eliminating many intermediate steps like downloading the structure in formats like CIF or XYZ and converting it to TURBOMOLE format, thereby streamlining the process of getting started with Riper.
  - Converting structure files in popular formats like CIF, POSCAR, XYZ, and Quantum ESPRESSO^3,119,120^ to the format expected by Riper, simplifying the process of working with diverse data sources, making a switch from other packages to TURBOMOLE convenient.
  - Generating input parameters and keywords for RT-TDDFT calculations, which are typically more complex to set up manually, as they require the user to specify various parameters such as electric field type, strength, frequencies, evolution time, time step, time evolution algorithm, and so on.^18^
  - Generating input for band structure calculations can be cumbersome and error-prone when done manually. Riper-Tools simplifies this by automatically determining high-symmetry *k*-points based on ref 121, as facilitated by Pymatgen and ASE, and generating the necessary input for Riper.
  - The web interface also facilitates the creation of input files for DFT-based embedding calculations. The users can specify the atoms to be considered as the active and environment subsystems, as well as the embedding method and other parameters conveniently, and download the input and coordinate files.
- Output Analysis
  - Parsing and plotting of RT-TDDFT absorption spectra from output files.
  - Electron density cube file operations, including addition, subtraction, multiplication, translation, integration, and planar averaging, enabled by the CubeToolz^122^ Python library.
  - Density of states (DOS) plotting capabilities after DOS calculations.
  - Band structure plotting after a band structure calculation.
  - Extraction and visualization of key results (e.g., energies at each iteration, structure) from Riper output files.
  - Visualization of chemical systems from coord files using Py3Dmol.^112,113^

- Modeling
  - Supercell creation for large systems or defect simulations.
  - Structure translation within the unit cell for precise atomic positioning.
  - Adsorbate placement on surfaces to study surface-adsorption phenomena.
  - Cell packing with a molecule to create specific molecular densities.
  - Surface orthogonalization, converting nonorthogonal surface cells (e.g., hBN monolayer) into orthogonal cells for ease of modeling.

These features collectively make Riper-Tools a valuable asset for researchers using the Riper module of TURBOMOLE. By providing a user-friendly interface and automating many common tasks, Riper-Tools enhances the accessibility and efficiency of performing DFT calculations, enabling users to focus on exploring chemical and materials properties. It is also worth mentioning that in addition to a detailed documentation and text tutorials, the web application is accompanied by hands-on YouTube tutorials on various aspects of quantum chemistry calculations with TURBOMOLE, created by one of the authors.^123^ It is also mentioned in passing that the TURBOMOLE coord files of both molecular and periodic systems can now be visualized using CrysX-3D Viewer,^124^ a cross-platform visualization application that runs on Linux, Mac, Windows, and Android devices.

## Applications

### Rashba Splittings and the Importance of the Current Density

Rashba splitting occurs due to the momentum-dependent splitting of spin bands in low-dimensional condensed matter systems, as, for example, in the prominent transition-metal dichalcogenide monolayers of MoCh_2_ and WCh_2_ (Ch = S, Se, Te) in the hexagonal (2H) phase. Here, time-reversal symmetry holds, and ϵ_+**k**_^↑^ = ϵ_–**k**_^↓^, however, space-inversion symmetry does not hold and hence ϵ_+**k**_^↑^ = ϵ_–**k**_^↑^ is not generally true. Therefore, the energies of the spin-up and spin-down states at a general *k*-point, e.g., at the *K*-point may differ.

As presented in ref 16, current-dependent DFT plays an important role when describing these Rashba splittings, if of course the underlying functional is at least of kinetic-energy dependent metaGGA quality. As outlined above, any metaGGA that depends on the kinetic-energy density should employ the current-dependent form, in the case of spin–orbit coupling (SOC) being considered variationally. As this is *a priori* required for the accurate description of Rashba effects, any variational calculation of the latter will be current-dependent, leading to a considerable error in the obtained splittings if the spin current density is neglected. Exemplary results are listed in Figure 2. For comparison, we also include results with the range-separated GGA hybrid HSE06,^49^ where the Fock exchange contribution uses all density matrix part or excludes the ones associated with the spin current densities **Y**. Rashba splittings are shown for two different basis sets, namely the Karlsruhe dhf-TZVP-2c bases optimized for molecular calculations^125^ and the pob-TZVP-rev2 basis optimized for periodic calculations.^126–128^ All calculations are carried out with the latest development version of TURBOMOLE.^17^ As is evident from the figure, the choice of basis set strongly affects the Rashba splittings of the tungsten-based monolayers.

**Figure 2:**
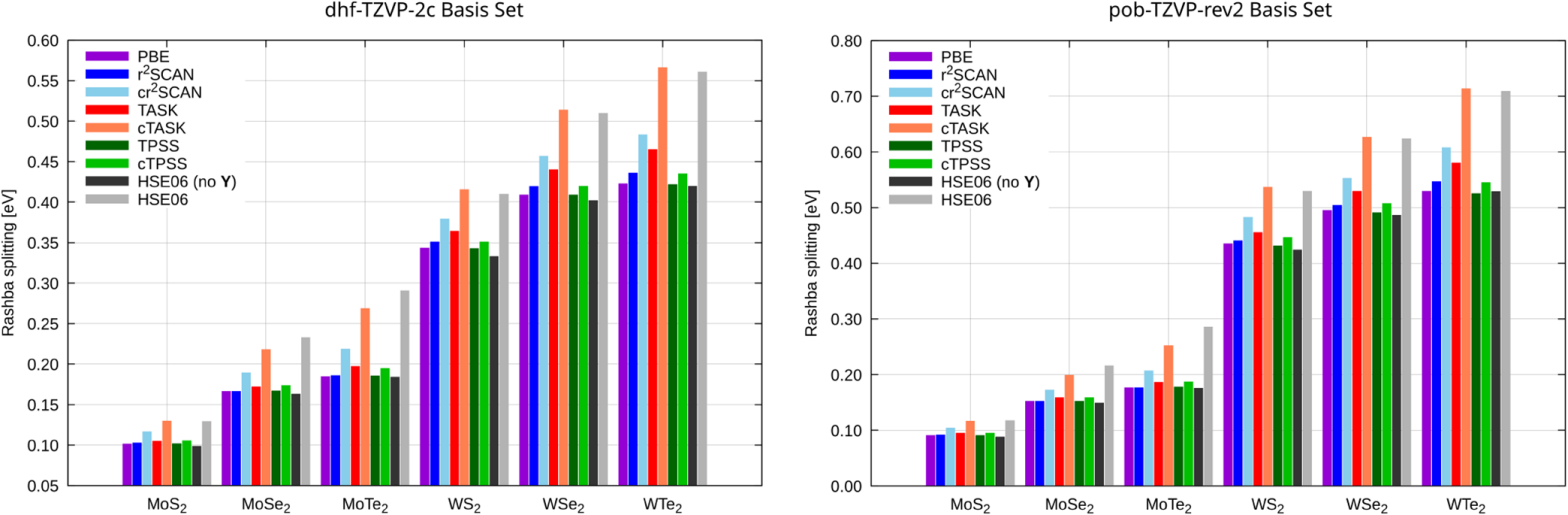
Rashba splittings of the valence band at the *K*–point for transition-metal dichalcogenide monolayers at the 1c DFT, 2c DFT, and 2c CDFT level with the PBE,^129^ TPSS,^130^ r^2^SCAN,^131,132^ TASK,^133^ and HSE06 ^49^ functionals. The application of the CDFT formalism is indicated by a “c” for the functional acronym. All values in eV. Left panel: dhf-TZVP-2c basis set.^125^ Right panel: pob-TZVP-rev2 basis set.^126–128^ All calculations use a 33 × 33 *k*-mesh and Dirac–Fock ECPs for Mo (28), Se (10), Te (28), and W(60).^134–136^ Thresholds, RI auxiliary basis set, and computational details are the same as in refs 16,17 .

Especially for the strongly current-dependent density functional approximations r^2^SCAN^131,132^ and TASK,^133^ changes of up to 25% are observed upon the neglect or inclusion of the current density. These strong effects can be attributed to the exchange and correlation enhancement factors employed in the construction of these functionals. Both TASK and r^2^SCAN use the same iso-orbital indicator α to interpolate between iso-orbital and uniform electron gas limits. This iso-orbital indicator α, itself being constructed from kinetic-energy densities,^137^ automatically yielding this dependence. Still, the effect observed for WSe_2_ with r^2^SCAN is considerably smaller than predicted in ref 138. This is likely caused by the definitions of τ differing by a factor of 2.^78^ We chose the definition that ensures gauge invariance within a static magnetic field^66^ and consistency within the TDDFT frameworks^78^ as shown by a comparison of RT-TDDFT and linear-response TDDFT in the Supporting Information. Obviously, TASK has been constructed with a steeper slope of α, leading to significantly more pronounced effects. Overall, the absolute magnitude of the effect of current densities on Rashba splitting depends on the material, although the relative effect is of the same order of magnitude for each material. This outlines that for significant Rashba effects, also the impact of the current density will be significant and therefore must not be neglected. As further demonstrated in refs 16,17 , this is not the case for simple band gaps, which are rather independent of the current density.

### Band Structure of the MoSe_2_ Monolayer

To further illustrate the capabilities of Riper, we present band structure calculations for monolayer MoSe_2_ (2H phase). As discussed in the last paragraph, this system is known for its significant valence-band splitting at the *K* point, which arises from SOC and the lack of inversion symmetry. We calculate the band structure using the PBE (GGA),^129^ HSE06 (range-separated hybrid),^49^ and SCAN0 (hybrid metaGGA)^139^ functionals, with spin–orbit coupling included. The calculations are performed using the pob-TZVP-rev2 basis set,^126,127^ Dirac–Fock ECP28 for Mo,^135^ and a 33 × 33 *k*-point mesh for sampling the Brillouin zone (large DFT grid size m5). The monolayer geometry is obtained from ref 140. Figure 3 compares the band structures obtained with PBE, HSE06, and SCAN0. The band gaps computed using PBE (1.39 eV) and HSE06 (1.80 eV) with Riper are in excellent agreement with plane-wave benchmark values (PBE: 1.35 eV; HSE06: 1.75 eV).^141^ The SCAN0 functional yields a band gap of 2.46 eV, which closely matches the reference *GW* value of 2.41 eV.^141^ With cSCAN0, a gap of 2.47 eV is obtained. The band structures without SOC are also shown with gray lines in Figure 3, to underscore the importance of the proper treatment of SOC for capturing the valence-band splitting at the *K*–point.

**Figure 3:**
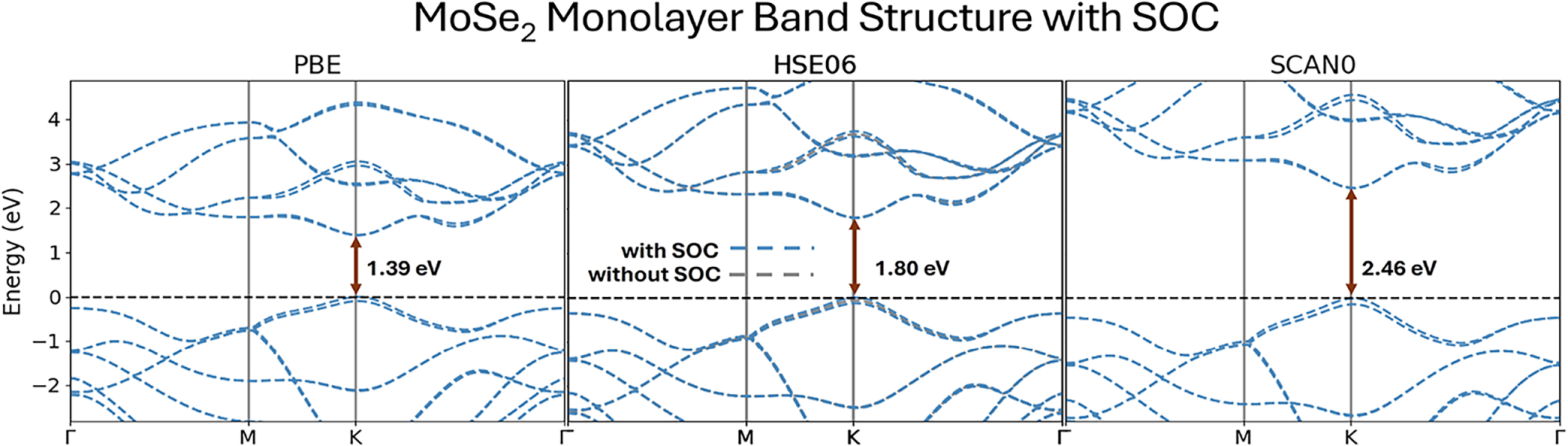
Band structures of monolayer MoSe_2_ computed using different XC functionals: PBE, HSE06, and SCAN0. The direct band gap at the *K*–point is indicated for each method, showing a significant increase in the gap from 1.39 eV (PBE) to 2.46 eV (SCAN0), highlighting the role of hybrid and metaGGA functionals in improving electronic structure predictions.

As mentioned earlier, the Riper-Tools web application can be used to streamline band structure calculations, such as the one presented here. The procedure is described briefly as follows:

1. **Upload** CIF **File.** After uploading the crystal structure (in CIF format) to the web app, ASE and pymatgen are used to identify the Bravais lattice and to reduce the cell to its primitive form if necessary (via spglib).
2. **2D Flag and Path Generation.** For 2D materials such as monolayer MoSe_2_, a dedicated flag generates an appropriate high-symmetry path in the 2D Brillouin zone (e.g., Γ → *M* → *K* → Γ).
3. **Input Snippet Creation.** The tool then produces the necessary snippet for the $kpoints section of the control file in Riper. An example for MoSe_2_ is 
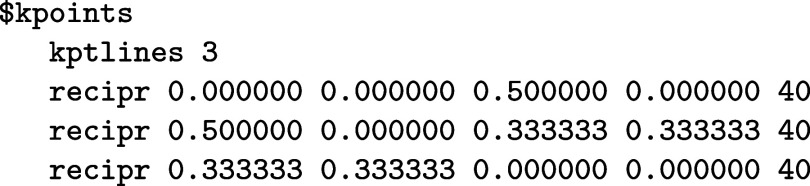
 This can be directly copied to the control file, saving the user from having to manually define the high-symmetry path.

Once the calculation is complete, a Python script (available in the GitHub repository of Riper-Tools^142^ can be used to parse the output and plot the resulting band structure with matplotlib (as the one shown in Figure 3). This end-to-end workflow, from structure file to plotted band diagram, significantly simplifies what can otherwise be a tedious process.

### Magnetic Transition of a Pt Chain with 2c DFT

One dimensional Pd and Pt chains are well studied systems to illustrate the transition of a nanomaterial from a closed-shell configuration to an open-shell configuration. For a small Pt–Pt distance, the closed-shell or nonmagnetic electronic configuration is lower in energy, while the open-shell state becomes the ground state with increasing distance.^143–147^ Figure 4 outlines this behavior at the spin–orbit PBE^129^ and TPSS^130^ levels. Here, two Pt atoms are placed in the unit cell, and a closed-shell solution is found for cell parameters of *d* = 4.0 to *d* = 4.8 Å. That is, an open-shell guess converges into a closed-shell solution with a vanishing spin magnetic moment. Additionally, the equilibrium structure is at *d* ≈ 4.8 Å with PBE and at *d* ≈ 4.7 Å with TPSS/cTPSS. The impact of the current density on the potential energy surface and relative energies is rather small.

**Figure 4:**
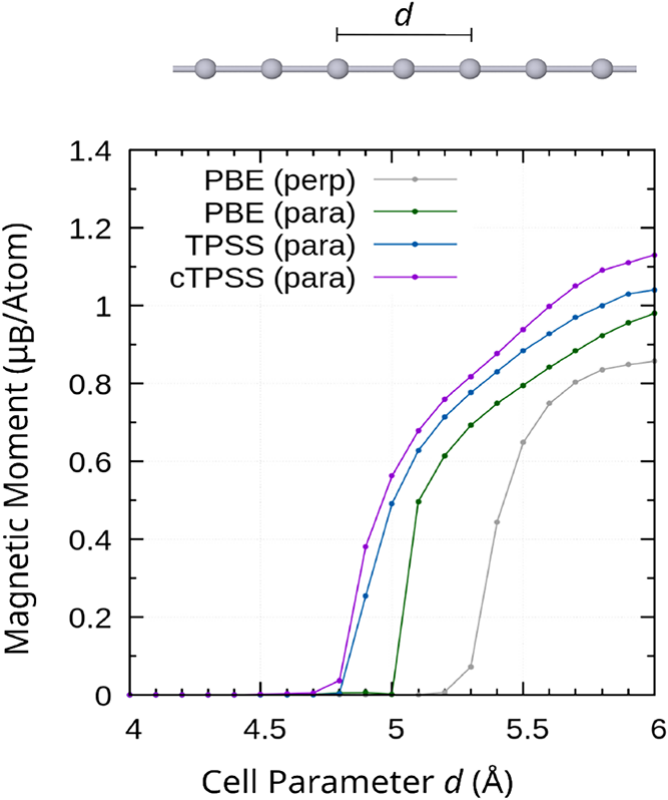
Magnetic moment in units of Bohr’s magneton μ_B_ per atom for the spin contribution of 2c canonical noncollinear DFT (PBE, TPSS) and CDFT (cTPSS) approach. Starting with *d* ≈ 4.8 Å, the open-shell solutions are energetically favored compared to the closed-shell solutions, and the spin alignment parallel (para) to the chain is preferred over the alignment perpendicular (perp) to the chain. The unit cell includes two Pt atoms. Results collected from refs 15,16 and plotted for this work. Picture of Pt chain reproduced from ref 16 under a CC BY license. Copyright 2024 the Authors.

For the open-shell configuration, the spin magnetization can be aligned parallel or perpendicular to the chain with a two-component formalism. Here, the energy for the parallel alignment is more favorable, and the transition to the open-shell state occurs at smaller cell parameters. Additionally, a large magnetic moment is observed. This moment increases from PBE to TPSS and cTPSS. Overall, these results are in excellent qualitative agreement with previous studies based on plane-wave approaches at the LDA and PBE level.^146^

### Adsorption Energy of H_2_O on LiH (001) from WFT-in-DFT

Predicting adsorption energies is important in understanding surface interactions in heterogeneous catalysis, materials science, and environmental chemistry. As shown in refs 21,148 , KEDF-based molecule-in-periodic embedding coupled with correlated WFT methods can be used to predict the adsorption energy of molecules highly accurately. For water (H_2_O) adsorption on LiH (001) (Figure 5), the LDA adsorption energy (−474 meV)^148^ significantly overestimates the periodic MP2 reference (−219 meV),^149^ highlighting LDA’s overbinding tendency.

**Figure 5:**
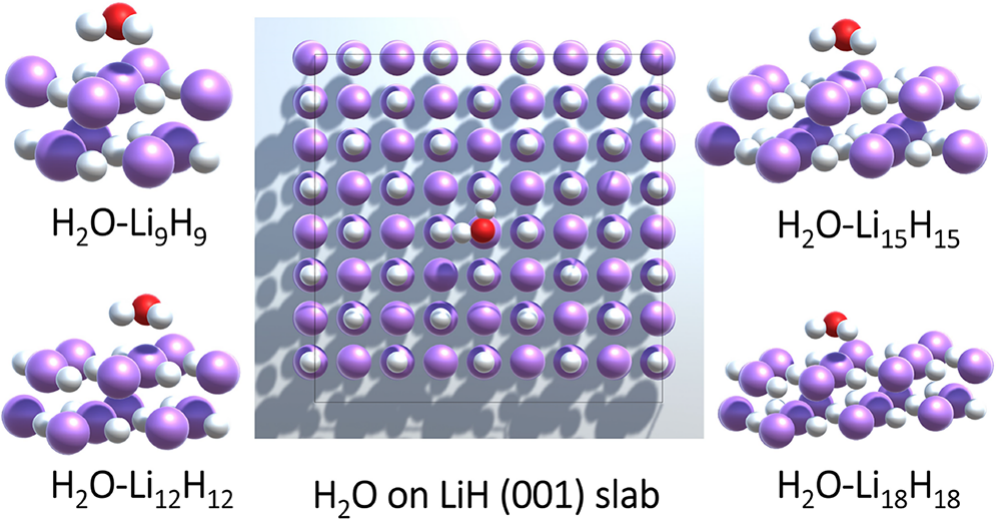
Graphical representations of the adsorption configuration of a water (H_2_O) molecule on a two-layer LiH (001) slab (center panel) and the different fragment sizes used for WFT-in-DFT embedding (surrounding images) Lithium atoms are shown in purple, oxygen in red, and hydrogen in white. Adapted with permission from ref 148 under a CC BY-NC-SA license. Copyright 2024 the Authors.

For WFT-in-DFT embedding calculations, the active subsystem is defined to include the water molecule and nearby LiH atoms, while the rest of the slab is treated as the environment subsystem. By employing KEDF-based molecule-in-periodic embedding, the MP2-in-LDA adsorption energy is computed for different fragment sizes (H_2_O–Li_*n*_H_*n*_ with *n* = 9, 12, 15, and 18 - Figure 5), to assess convergence with respect to cluster size.^148^

For the smallest fragment (H_2_O – Li_9_H_9_), the MP2-in-LDA adsorption energy is −191 meV, converging to −220 meV for larger fragments (H_2_O–Li_15_H_15_ and H_2_O–Li_18_H_18_), in excellent agreement with a periodic MP2 adsorption energy of −219 meV as reported in ref 149.

Interestingly, using the PBE functional, the adsorption energy is predicted to be −212 meV, which is already quite close to the MP2 reference. Applying MP2-in-PBE embedding for the largest fragment further refines the result to −215 meV, demonstrating that the error-compensation strategy of embedding (see Section 6) can work in both directions—reducing or slightly enhancing binding as needed.

These results underscore the potential of DFT-based embedding in accurately capturing the local correlation effects in adsorption studies. By selectively applying wave function-based corrections to key regions of interest, this approach enables computationally efficient yet highly accurate adsorption energy predictions. Furthermore, the computational time is significantly shorter than a full periodic WFT calculation, only taking as long as a DFT calculation of the total system plus a molecular WFT calculation of the active subsystem. Such a framework can be extended to more complex surfaces and molecular adsorbates, making it a valuable tool for computational materials design and surface chemistry investigations.

### Optical Gaps of Ionic Solids from *GW*/BSE-in-DFT

Accurate optical property predictions in ionic solids are essential for electronic and optoelectronic applications. *GW*/BSE provides highly accurate excitation energies of periodic systems.

As reported in ref 109, the *GW*/BSE-in-DFT method employing a KEDF-based embedding potential achieves a high level of accuracy in predicting optical gaps for ionic solids such as MgO, CaO, LiF, NaF, KF, and LiCl, with a mean absolute error (MAE) of just 0.38 eV compared to experimental values. The computational efficiency of the method is notable; for instance, the *GW*/BSE-in-DFT calculation for the largest LiF cluster required only 138 s. The excitation energies calculated by using *GW*/BSE-in-DFT also demonstrate quick convergence with respect to cluster size. The method’s versatility is demonstrated by its successful application to calculating the optical gap of 2D MgCl_2_ and the excitation energy of an oxygen vacancy in MgO. The *GW*/BSE-in-DFT results exhibit excellent agreement with both experimental and reference periodic *GW*/BSE values. Notably, the discrepancy between *GW*/BSE-in-DFT and periodic *GW*/BSE decreases as the ionic character of the solid increases from LiF to NaF to KF. It is also shown that the surrounding environment significantly impacts the excitation energies of the ionic clusters, with the embedding process leading to excitation energies that are consistent across different cluster sizes and significantly higher than those of the isolated clusters. Table 2 summarizes the key results obtained from *GW*/BSE-in-DFT calculations for the largest clusters of each material.

**Table 2:** Comparison of *GW*/BSE-in-DFT Calculated Optical Gaps with Experimental and Periodic (PBC) *GW*/BSE Values^a^

material	*G*W/BSE-in-DFT	experiment	*G*W/BSE (PBC)
MgO	7.71	7.70 ^150^	8.10 ^151^
CaO	7.43	6.90 ^150^	6.90 ^152^
LiF	12.09	12.61 ^153^	12.99 ^154^
NaF	9.82	10.71 ^153^	10.64 ^154^
KF	9.50	9.76 ^155^	9.76 ^154^
LiCl	8.94	8.90 ^156^	8.80 ^157^

a All results in eV. Adapted with permission from ref 109. under a CC BY license. Copyright 2024 the Authors.

The implementation of *GW*/BSE-in-DFT within TURBOMOLE provides an efficient tool for studying the optical properties of ionic materials. The method accurately captures environmental effects, making it a reliable approach. The method can potentially be applied to other systems with noncovalent interactions.

### HHG in Water Cluster Using RT-TDDFET

HHG in molecular systems provides valuable insights into the electron dynamics under intense laser fields. The HHG spectrum is computed by Fourier transforming the time-dependent dipole acceleration μ̈(t), which corresponds to the second derivative of the induced dipole moment with respect to time. The resulting power spectrum *P*(ω) is given by

83 $$P\left(\omega \right)={\left|\frac{1}{t_{f}-t_{i}}\int_{t_{i}}^{t_{f}}W\left(t\right)\frac{d^{2}\mu^{\mathrm{ind}}\left(t\right)}{dt^{2}}e^{-i\omega t}\mathrm{dt}\right|}^{2}$$

where *W*(*t*) is the Hann window function used to minimize edge effects due to the finite simulation time, and [*t*_*i*_,*t*_*f*_] denotes the total propagation interval. The induced dipole moment is defined as

84 $$\mu_{j}^{\mathrm{ind}}\left(t\right)=\mu_{j}\left(t\right)-\mu_{j}^{0},\mu_{j}\left(t\right)=\mathrm{Tr}\left[M^{j}\cdot D\left(t\right)\right]$$

with **M** ^*j*^ and **D**(*t*) denoting the dipole moment (eq 79) along direction *j* = *x*, *y*, *z* and time-dependent density matrices, respectively. This formalism is commonly used for HHG in molecular systems.^158–161^

It is shown in ref 148 that the HHG spectrum of an isolated water molecule under an intense laser pulse, simulated using RT-TDDFT, differs significantly from that of a water molecule embedded in a (H_2_O)_44_ cluster, using the RT-TDDFET implementation described previously. KEDF-based RT-TDDFET successfully accounts for environmental effects while maintaining computational feasibility. A similar study has also been conducted in ref 87, but with a much smaller (H_2_O)_5_ cluster.

For both the isolated and embedded water molecule, the HHG spectrum exhibits distinct perturbative, plateau, and cutoff regions (see Figure 6), consistent with Corkum’s three-step model.^162^ The ionization potential (*I*_*p*_) is calculated to be 12.34 eV, in good agreement with the experimental value of 12.62 eV, and the cutoff energy of 22.3 eV (18th harmonic) follows the expected cutoff law. Harmonic splitting is observed due to resonances with optical absorption peaks, highlighting the role of the electronic structure in the HHG process.

**Figure 6:**
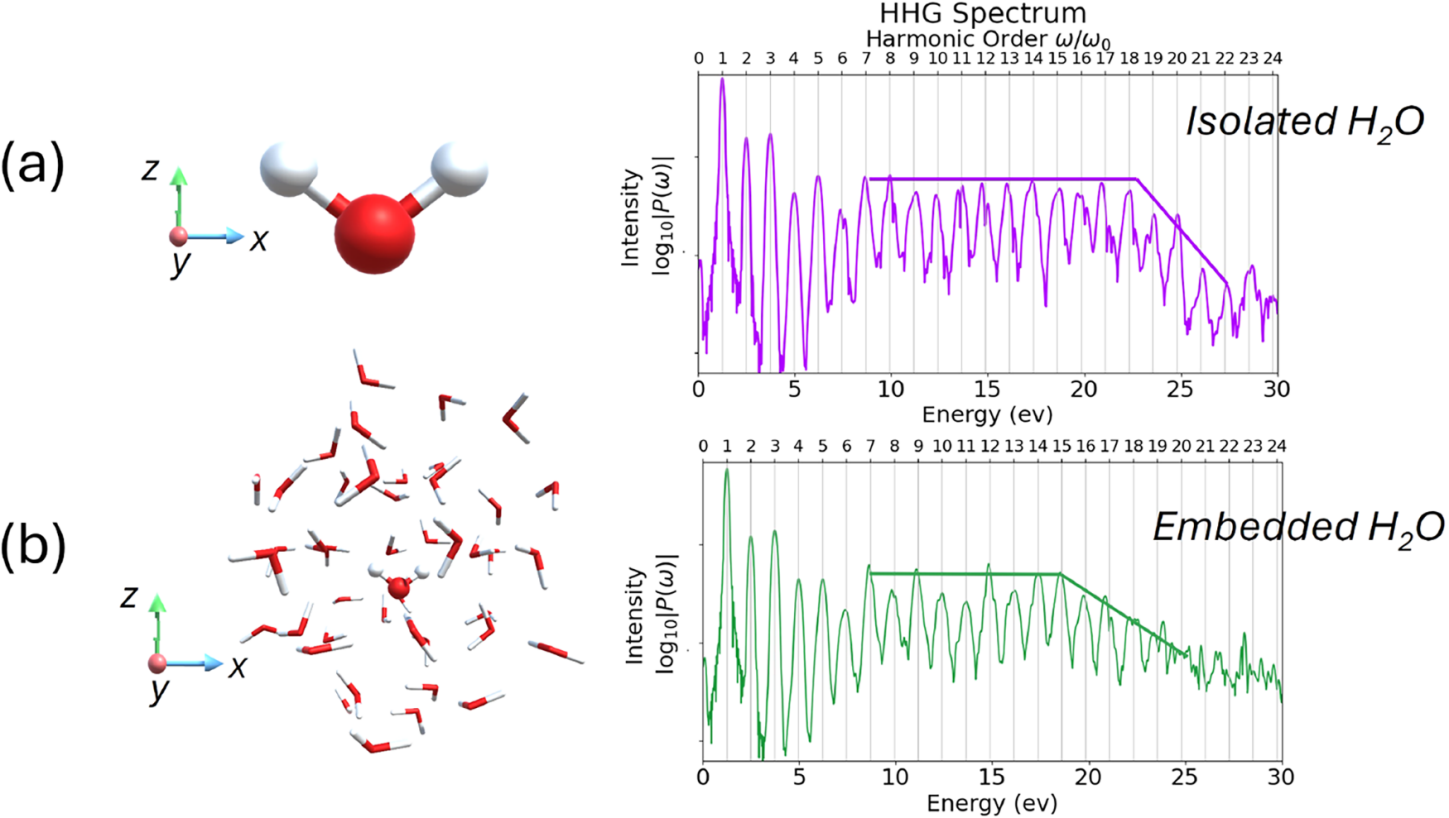
HHG spectra of an isolated H_2_O molecule (a) and an H_2_O molecule embedded in a (H_2_O)_44_ cluster (b), obtained using RT-TDDFT and KEDF-based RT-TDDFET, respectively. The spectra exhibit characteristic perturbative, plateau, and cutoff regions, with a redshift in the HHG cutoff observed for the embedded molecule, indicating a reduction in ionization potential due to environmental screening effects. Adapted with permission from ref 109 under a CC BY-NC-SA license. Copyright 2024 the Authors.

For the embedded water molecule, a redshift in the HHG cutoff to the 15th harmonic (18.6 eV) is observed, indicating a ∼ 2 eV reduction in *I*_*p*_. This shift is attributed to environmental screening effects due to hydrogen bonding and aligns with previous theoretical and experimental studies on ionization energy shifts in water clusters.^148,163^ These results demonstrate that KEDF-based RT-TDDFET provides an accurate and computationally efficient framework for modeling nonlinear optical phenomena in complex environments. Future work could extend this approach to dynamic hyperpolarizability in molecular systems.

### *Ab Initio* Molecular Dynamics of Liquid Water

Water’s fundamental role in life and chemistry is underpinned by its surprisingly complex behavior despite its simple structure. Investigating its unusual properties, particularly in the liquid phase, requires accurate molecular dynamics simulations. Unlike classical molecular dynamics which uses empirical potentials, *ab initio* molecular dynamics (AIMD) directly computes the forces between atoms using quantum mechanical methods such as DFT at each simulation step. This fundamental difference allows AIMD to model phenomena where the electronic structure adapts to nuclear motion and chemical bonds are dynamic, making AIMD uniquely suited for simulating chemical processes involving water. In this context, to further demonstrate the flexibility of the Riper module in modeling periodic systems, we perform a short, demonstrative all-electron AIMD simulation of bulk liquid water with PBC and obtain insights about its structure.

AIMD simulations are performed using a cubic simulation cell with a side length of 12.4198 Å containing 64 water molecules (generated using Riper-Tools), similar to previous studies.^164,166^ The optimized geometry is provided in the Supporting Information. The pob-DZVP-rev2 basis set^127^ is employed along with the universal auxiliary basis set for DF.^167^ A custom ASE calculator is created for Riper and used to carry out DFT-based AIMD simulation in the *NVT* ensemble at a temperature of 300 K, employing the PBE functional with the D3 dispersion correction^168^ (PBE+D3)­(see Supporting Information for the Python script). The temperature is controlled using a canonical sampling through velocity rescaling (CSVR) thermostat^169^ for a total of 5 ps (time step of 0.5 fs), with the initial 2 ps for equilibration, and the subsequent 3 ps used for the calculation of the oxygen–oxygen radial distribution function (RDF). The complete trajectory and the corresponding movie are provided in the Supporting Information.

Figure 7 shows the calculated oxygen–oxygen (O–O) RDF (*g*_O–O_(r)) compared with another theoretical study utilizing PBE+D3 ^164^ as well as X-ray scattering experiments.^165^ The calculated RDF exhibits the first peak at *r*_1_ = 2.75 Å with peak height *g*_O–O_^max^(*r*) = 3.447. These values are in excellent agreement with PBE+D3 results (*r*_1_ = 2.75 Å and *g*_O–O_^max^(*r*) = 3.336) in ref 164, despite the relatively short simulation time. These values also compare reasonably well with the experimental data available in the literature. The simulated *r*_1_ of 2.75 Å, is quite close to the 2.73 Å,^165,170^ and 2.80 Å,^171^ values reported in experiments, suggesting a reasonable representation of the average oxygen–oxygen distance. The experimental *g*_(O–O)_^max^(*r*) values are reported to range between 2.57 and 3.00 under ambient conditions.^165,170,171^ The peak height predicted by PBE+D3 is slightly higher, which is a known tendency for liquid water simulated with common DFT approximations such as PBE+D3 at 300 K without additional corrections or elevated temperatures to implicitly account for nuclear quantum effects. This overestimation of the first peak height often indicates an overstructuring of the simulated water, where the tetrahedral hydrogen bond network is more pronounced than that in the experiment.

**Figure 7:**
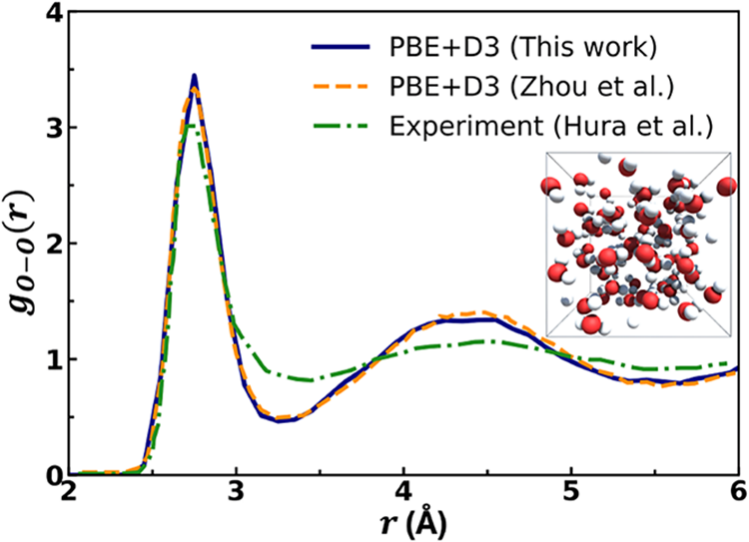
O–O radial distribution function *g*_O–O_(*r*) calculated using PBE+D3 compared with a similar calculation in ref 164 and experimental data.^165^ Inset shows the snapshot at 5 ps.

The above result demonstrates that Riper is well-suited for AIMD simulations of periodic systems. Additionally, instead of the current all-electron setup, in the future, one could explore the use of pseudopotentials to further enhance the computational efficiency.

## Benchmarking and Performance Analysis for Condensed Matter Hybrid-DFT Calculations

In this section, we demonstrate the capability of the Riper module to perform periodic hybrid-functional DFT calculations for exceptionally large systems with up to 2,736 atoms and 43,200 basis functions employing desktop workstations.

For a comprehensive assessment of performance, benchmark calculations are carried out on six chemically and structurally diverse condensed matter systems: porous aluminum terephthalate (MIL-53),^172^ faujasite, metal–organic framework-5 (MOF-5),^173,174^ two differently sized 2D SiO_2_ slabs, and bulk (3D) Si and MgO crystalline systems. Their structures are visualized in Figure 8. These systems are selected to reflect realistic computational workloads and encompass a broad spectrum of dimensionalities and bonding environments, ranging from extended porous 3D frameworks and dense crystalline solids to layered 2D materials.

**Figure 8:**
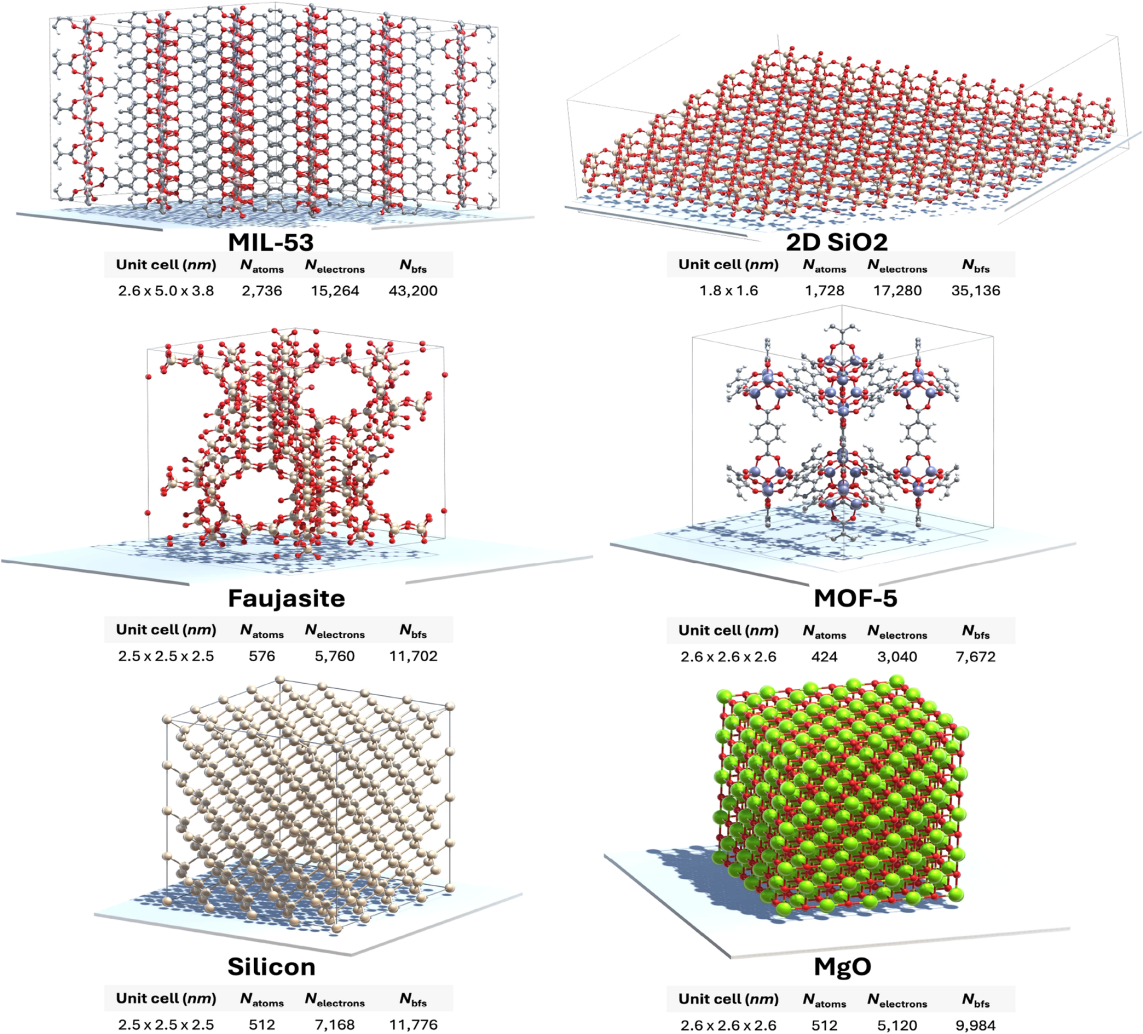
Graphical depiction of periodic systems used in benchmarking the Riper module, including MOFs (MIL-53, MOF-5), a zeolite (faujasite), layered 2D SiO_2_, and 3D dense crystals (Si and MgO). For each system, unit cell dimensions, number of atoms, electrons, and Gaussian basis functions are provided. Visualization generated using CrysX-3D Viewer.^124^

MIL-53 is a flexible MOF composed of [M–OH]­chains linked by terephthalate ligands, featuring 1D diamond-shaped pores that exhibit a reversible “breathing effect” in response to external stimuli. MOF-5 is a cubic metal–organic framework composed of Zn_4_O clusters and benzodicarboxylate linkers, notable for its high surface area and early application in hydrogen storage research. Faujasite represents a classical zeolite structure, while the 2D SiO_2_ slabs provide insights into layered material performance at different system sizes. The Si and MgO crystals represent prototypical bulk (3D) crystalline systems with fundamentally different bonding characteristics: covalent semiconductor and ionic insulator, respectively, allowing for direct comparison of computational efficiency across distinct electronic structure types.

All calculations are performed using the hybrid B3LYP functional^175^ along with the pob-TZVP-rev2 basis set^126,127^ specifically optimized for periodic systems. Universal auxiliary basis is employed for DF, although it was originally developed for molecular systems and may not be fully optimized for periodic calculations.^167^ All calculations maintained a convergence criterion of 10^–6^ E_h_ for the SCF energy (medium DFT grid size m3). SCF is carried out at the Γ-point only, and no space-group symmetry is exploited. Computations are executed on a high-performance Threadripper Pro 5995WX system with 64 cores and 512 GB memory. The reported wall times represent averages from 10 SCF iterations to ensure reliable benchmarking results.

Table 3 presents a comprehensive analysis of the wall time and component breakdown for SCF iterations across the test systems. For the MIL-53 system (2736 atoms and 3D PBC), which represents the largest system considered in this study, each SCF iteration takes 146 min on average. This is followed closely by the large 2D SiO_2_ slab (1728 atoms and 2D PBC), requiring 129.8 min per iteration. It is worth emphasizing that periodic hybrid-DFT calculations of this magnitude are quite rare in the literature and are generally restricted to massively parallel computing environments with thousands of cores.^176^ The fact that such calculations can be performed on a standard workstation underscores the exceptional efficiency of the Riper module and makes these demanding simulations more accessible to researchers with modest hardware.

**Table 3:** Wall Time per SCF Iteration (Minutes) and Component Breakdown (Percent) for Various Systems; Hybrid B3LYP Functional and pob-TZVP-rev2 Basis Sets

system	*N*_atoms_	*N*_elec_	*N*_bf_ (cart)	Total (min)	**J** (%)	**K** (%)	XC (%)	diag (%)	misc (%)
MIL-53	2736	15264	43200	146.0	6.2	24.6	0.2	62.3	6.7
Faujasite	576	5760	11712	5.5	18.5	62.0	1.0	10.6	7.9
MOF-5	424	3040	7672	2.2	7.9	61.5	1.4	18.9	10.3
2D SiO_2_ (small)	144	1440	2928	0.6	19.0	67.0	3.0	7.7	3.3
2D SiO_2_ (large)	1728	17280	35136	129.8	2.5	25.9	0.1	65.9	5.6
3D Si	512	7168	11776	20.1	18.7	56.3	0.6	20.7	3.7
3D MgO	512	5120	9984	22.8	31.4	54.2	0.8	11.1	2.5

A detailed examination of the time distribution across computational components reveals interesting trends that highlight the module’s efficiency. For the largest systems (MIL-53 and large 2D SiO_2_), the total time required for KS matrix construction—comprising Coulomb (**J**), exchange (**K**), and XC contributions—constitutes less than 31% of the total wall time. This demonstrates the remarkable efficiency of the various algorithms implemented in the Riper module. For these large systems, the diagonalization step dominates the computational cost, consuming 62.3% and 65.9% of the total time for MIL-53 and large 2D SiO_2_, respectively. This is expected given the exceptionally large matrices resulting from the significant number of basis functions (43,200 for MIL-53 and 35,136 for large 2D SiO_2_).

The next most time-consuming component is the **K** matrix construction, which requires approximately 25% of the total time for the largest systems. Notably, the **J** matrix construction is highly efficient, taking only 6.2% for MIL-53 and 2.5% for large 2D SiO_2_. Even more impressive is the negligible time required for XC matrix construction (0.2% and 0.1% for MIL-53 and large 2D SiO_2_, respectively), which takes significantly less time than miscellaneous operations such as octree preparation, shell indices list construction, grid generation, and reading the initial guess from disk.

The computational profile changes significantly for medium-sized systems such as faujasite (3D PBC, 576 atoms), Si (3D PBC, 512 atoms), MgO (3D PBC, 512 atoms), and MOF-5 (3D PBC, 424 atoms), as well as the smaller 2D SiO_2_ system (2D PBC, 144 atoms). For these systems, the **K** matrix construction becomes the dominant computational bottleneck, consuming 54.2–67% of the total wall time, while diagonalization becomes less demanding, in contrast to MIL-53 and large 2D SiO_2_. However, even for these smaller systems, the XC matrix construction remains remarkably efficient, requiring only 1–3% of the total computation time.

A direct comparison between the Si and MgO supercells, both containing 512 atoms, reveals interesting computational differences, despite their similar system sizes. While both require similar wall times for SCF (∼20 min), the MgO system exhibits significantly higher Coulomb matrix (**J**) construction time (31.4% vs 18.7% for Si), nearly doubling the computational cost. This increased **J** time for MgO arises from the ionic nature of the Mg–O bonds, which creates a more challenging electrostatic environment with stronger and longer-range Coulomb interactions compared with the covalent Si–Si bonds. Conversely, the diagonalization step is more efficient for MgO (11.1% vs 20.7% for Si), likely due to the smaller basis set requirements (9,984 vs 11,776 basis functions), fewer electrons, and the more localized electronic structure of the ionic system, which leads to sparse matrices and faster diagonalization. Interestingly, faujasite with a similar system size as Si and MgO takes significantly less time (only 5.5 min) for a single SCF iteration. This is attributed to its porous nature, thereby requiring much fewer integral evaluations due to fewer overlapping basis functions.

Scaling behavior is another important aspect of computational efficiency. Comparing the small and large 2D SiO_2_ systems provides valuable insights into this. When increasing from 144 to 1728 atoms (a factor of 12), the total wall time increases from 0.6 to 129.8 min, indicating favorable subquadratic scaling overall. Examining individual components reveals even better scaling characteristics: the Coulomb term scales nearly linearly, increasing from 6.6 to 199 s, while the XC term demonstrates excellent linear scaling, growing from 1 to 11 s. The exchange term exhibits subquadratic scaling, increasing from ∼ 24 s for the 144-atom system to ∼ 2020 s for the 1728-atom system.

These benchmarking results demonstrate that the Riper module offers an efficient implementation for periodic DFT calculations, making it possible to study complex systems with thousands of atoms on modest computational hardware. The module’s favorable scaling characteristics and efficient matrix construction algorithms represent a significant advancement in making high-level quantum chemical calculations accessible for realistic periodic systems.

## Conclusions and Summary

The periodic DFT framework in TURBOMOLE, implemented through the Riper module, has undergone continuous development to improve its accuracy and efficiency for modeling extended systems. By employing Gaussian-type basis sets and resolution-of-identity techniques, the method provides a computationally efficient approach to studying periodic materials, surfaces, and interfaces, while maintaining a reasonable balance between cost and accuracy. Recent extensions have introduced additional functionalities, broadening the range of possible applications.

One of the most significant developments is the incorporation of spin–orbit coupling and relativistic effects within a two-component formalism. These features are particularly relevant for materials containing heavy elements, where relativistic interactions influence the electronic structure, magnetic properties, and optical behavior. The use of Gaussian-based real-space methods allows for an efficient treatment of these effects within the periodic DFT framework, making it possible to study systems that require a relativistic description.

Another notable improvement is the implementation of hybrid functionals and exact exchange methods for periodic calculations. Hybrid DFT provides better accuracy for properties such as band gaps and reaction energetics compared to conventional GGA functionals. The use of DF techniques and fast multipole methods has improved the computational feasibility of hybrid functionals in periodic systems, enabling more accurate predictions for semiconductors, insulators, and correlated materials.

Embedding methods have also been integrated, allowing for multiscale simulations that combine different levels of theory. Techniques such as FDE and PbE make it possible to treat specific regions of a system with higher accuracy, while keeping the rest of the environment at a lower computational cost. This is particularly useful for studying localized electronic states, adsorption phenomena, and heterogeneous interfaces.

The recent extension of RT-TDDFT to range-separated hybrid functionals broadens the scope of simulations of electronic dynamics. This approach allows for the study of light-induced excitations, nonlinear optical responses, and ultrafast electronic processes in periodic systems. The real-time propagation scheme avoids perturbative assumptions and enables direct modeling of nonequilibrium phenomena.

These methodological improvements enhance the versatility of periodic DFT calculations in TURBOMOLE, making them applicable to a wide range of problems in quantum chemistry, materials science, and condensed matter physics. Development efforts have focused on balancing accuracy, computational efficiency, and usability, ensuring that the methods are practical for studying electronic structure in extended systems.

Future improvements are expected to focus on further optimization of computational efficiency, better scalability for large systems, and integration of more advanced electron correlation methods, such as Møller–Plesset perturbation theory and coupled cluster approaches.

Overall, the recent developments in periodic DFT within TURBOMOLE provide a robust framework for studying a wide range of electronic and structural properties in extended systems. The implemented methods offer a balance between computational cost and accuracy, making them practical for both routine applications and more complex problems requiring a higher level of theory.

## Supplementary Material





## Notes

Optimized geometry of bulk liquid water used for AIMD simulations and the AIMD trajectory; Python script for performing AIMD with Riper; geometries of the systems used for performance benchmarking (ZIP)
Detailed description of RT-TDDFT with current-dependent and hybrid functionals (PDF)
