# Supplementary material for: Density Functional Theory for Molecular and Periodic Systems in TURBOMOLE: Theory, Implementation, and Applications
Source: J Phys Chem A. 2025 Sep 23;129(39):9062–83. doi: 10.1021/acs.jpca.5c02937 (PMC12498508; doi:10.1021/acs.jpca.5c02937)
Supplement: Supplementary file 2 [file jp5c02937_si_002.pdf]

Supporting Information: Density Functional Theory for Molecular and Periodic Systems in TURBOMOLE: Theory, Implementation, and Applications

Manas Sharma,[†,‡] Yannick J. Franzke,[†,¶] Christof Holzer,[§,‖] Fabian Pauly,[⊥] and Marek Sierka[*,†]

†*Otto Schott Institute of Materials Research, Friedrich Schiller University Jena, Löbdergraben 32, 07743 Jena, Germany*

‡*Department of Chemical Engineering, Indian Institute of Science, Bengaluru, Karnataka, 560012, India*

¶*Institute of Nanotechnology, Karlsruhe Institute of Technology (KIT), Kaiserstr. 12, 76131 Karlsruhe, Germany*

§*Institute of Theoretical Solid State Physics, Karlsruhe Institute of Technology (KIT), Wolfgang-Gaede-Str. 1, 76131 Karlsruhe, Germany*

‖*Insitute for Quantum Materials and Technologies, Karlsruhe Institute of Technology (KIT), Kaiserstr. 12, 76131 Karlsruhe, Germany*

⊥*Institute of Physics and Center for Advanced Analytics and Predictive Sciences, University of Augsburg, Universitätsstr. 1, 86159 Augsburg, Germany*

E-mail: marek.sierka@uni-jena.de

Contents

S1 RT-TDDFT with Current-Dependent metaGGAs S-2

S1.1 Fundamental Framework S-2

S1.2 Current-Dependent Exchange-Correlation Potential S-3

S1.3 Pilot Applications S-5

S1.4 Molecular Structures S-8

References S-10

S1 RT-TDDFT with Current-Dependent metaGGAs

Real-time time-dependent density functional theory (RT-TDDFT) offers an alternative to the more established linear-response TDDFT (LR-TDDFT) formalism. Herein, we will briefly outline the basic framework and its extension to current-dependent meta-generalized gradient approximations (metaGGAs). The reader is referred to refs. S1 and S2 for a comprehensive review on RT-TDDFT.

S1.1 Fundamental Framework

In RT-TDDFT, the time evolution of the electron density and the Kohn–Sham orbitals is computed by solving the time-dependent Kohn–Sham equations

$$\frac{\partial \psi_p(\mathbf{r},t)}{\partial t} = \left[-\frac{1}{2}\nabla^2 + \mathrm{v}_{\mathrm{eff}}\left[\rho\right](\mathbf{r},t)\right]\psi_p(\mathbf{r},t) \tag{1}$$

where $\mathrm{v}_{\mathrm{eff}}$ is the effective potential which is modeled with a given density functional approximation. The electron density is calculated with the Kohn–Sham orbitals ψ_p and their occupation numbers f_p according to

$$\rho(\mathbf{r},t) = \sum_p f_p |\psi_p(\mathbf{r},t)|^2 \tag{2}$$

In the linear combination of atomic orbitals (LCAO) ansatz the Kohn–Sham orbitals read

$$\psi_p(\mathbf{r},t) = \sum_\mu C_{\mu p}(t)\mu(\mathbf{r}) \tag{3}$$

where μ is a real basis function and $C_{\mu p}$ denotes the corresponding complex expansion coefficient. Within this ansatz, the density matrix $\mathbf{D}$ becomes the decisive quantity as it represents the time evolution of the electron density *via*

$$\rho(\mathbf{r},t) = \sum_{\mu\nu} D_{\mu\nu}(t)\mu(\mathbf{r})\nu(\mathbf{r}) \tag{4}$$

$$D_{\mu\nu}(t) = \sum_p f_p C^*_{\mu p}(t) C_{\nu p}(t) \tag{5}$$

Therefore, the dynamics is available from the von-Neumann equation

$$\mathrm{i}\frac{\partial \mathbf{D}(t)}{\partial t} = [\mathbf{F}(t), \mathbf{D}(t)] \tag{6}$$

where $\mathbf{F}(t)$ denotes the time-dependent Kohn–Sham matrix which also depends on the density matrix *via* the two-electron Coulomb and exchange-correlation matrix. The molecular system is perturbed from its initial state by the application of an electric field describing a laser pulse of an experimental setup. In the electric dipole approximation, the electric field $\mathbf{E}$ is assumed to be uniform over the complete molecule, i.e. $\mathbf{E}(\mathbf{r},t) = \mathbf{E}(t)$, and therefore the electric

field simply leads to an additional term

$$\mathbf{F}^{\mathrm{RT}}(t) = \mathbf{F}^{\mathrm{KS}}(t) + \mathbf{F}^{\mathrm{E}}(t) \tag{7}$$

Here, $\mathbf{F}^{\mathrm{KS}}$ denotes the molecular Kohn–Sham matrix

$$\mathbf{F}^{\mathrm{KS}}(t) = \mathbf{h}(0) + \mathbf{J}(t) + \mathbf{X}(t) \tag{8}$$

including the time-independent one-electron Hamiltonian $\mathbf{h}$, the Coulomb term $\mathbf{J}$, as well as the exchange-correlation matrix $\mathbf{X}$. $\mathbf{F}^{\mathrm{E}}$ describes the perturbation by the electric field based on the dipole operator in length gauge.

$$\mathbf{F}^{\mathrm{E}}_{\mu\nu}(t) = -\sum_{k=x,y,z} M^{k}_{\mu\nu} E_k(t) \tag{9}$$

$$M^{k}_{\mu\nu} = -\int \mu(\mathbf{r}) k \nu(\mathbf{r}) \ \mathrm{d}^3 r \tag{10}$$

The von-Neumann equation is solved with a Magnus expansion[S3,S4] and a unitary time evolution operator to conserve the idempotency of the density matrix. The initial second-order Magnus expansion reads

$$\mathcal{U}(\Delta t, 0) = \exp\left(-\mathrm{i}\Delta t \cdot \mathbf{F}^{\mathrm{RT}}(\Delta t/2)\right) \ \text{with} \ \mathbf{F}^{\mathrm{RT}}(\Delta t/2) \approx \mathbf{F}^{\mathrm{RT}}(0) \tag{11}$$

with $\mathbf{F}^{\mathrm{RT}}$ in the orthonormal molecular orbital basis. The exponential is evaluated with a diagonalization. The time evolution operator is then used to calculate the density matrix as

$$\mathbf{D}(\Delta t) = \mathcal{U}(\Delta t, 0)\mathbf{D}(0)\mathcal{U}^{\dagger}(\Delta t, 0) \tag{12}$$

This time propagation is then embedded into a self-consistent field (SCF) framework.[S5,S6] Acceleration is possible with the predictor-corrector scheme[S7] as implemented in refs. S5 and S8. The absorption spectra are finally computed from the dipole strength function[S8] and the Fourier transforms of the electric field and the induced dipole moment.[S9]

S1.2 Current-Dependent Exchange-Correlation Potential

A few comments on the fundamental framework are in order. The time evolution operator is complex, meaning that the density matrix for $t > 0$ will also be complex but Hermitian. Semilocal GGAs do not depend on the imaginary part of the density matrix, as the Kohn–Sham potential is purely real and symmetric (RS). However, the imaginary and antisymmetric (IA) part of the density matrix corresponds to the current density, i.e. electromagnetic perturbations induce a current density. That is, the kinetic energy density and the current density read

$$\tau = \frac{1}{2}\sum_{p} \left[\nabla \psi_p^*\right] \left[\nabla \psi_p\right] = \frac{1}{2}\sum_{\mu\nu} \mathbf{D}^{\mathrm{RS}} \left\{\left[\nabla \mu\right] \left[\nabla \nu\right]\right\} \tag{13}$$

$$\mathbf{j} = -\frac{1}{2}\sum_{\mu\nu} \mathbf{D}^{\mathrm{IA}} \left\{\left[\nabla \mu\right] \ \nu - \mu \ \left[\nabla \nu\right]\right\} \tag{14}$$

Note that we dropped the dependence on the time and space for brevity. Following the discussion in the main text, the generalized kinetic density has to be constructed according to

$$\tilde{\tau} = \tau - \frac{|\mathbf{j}|^2}{2\rho} \tag{15}$$

Therefore, metaGGAs should also depend on the imaginary antisymmetric density matrix. Note that the same holds for hybrid functionals due to the exact exchange integrals. In this current-dependent framework, the semilocal exchange-correlation energy at a given time follows as

$$\begin{aligned} E_{\mathrm{xc}} &= \int f_{\mathrm{xc}} \left[\rho(\mathbf{r}), \gamma(\mathbf{r}), \tau(\mathbf{r}), \mathbf{j}(\mathbf{r})\right] \ \mathrm{d}^3 r \\ &= \int g_{\mathrm{xc}} \left[\rho(\mathbf{r}), \gamma(\mathbf{r}), \tilde{\tau}(\mathbf{r})\right] \ \mathrm{d}^3 r \end{aligned} \tag{16}$$

with the gradient variable $\gamma(\mathbf{r}) = |\nabla \rho(\mathbf{r})|^2$. As usual, the potential for the Kohn–Sham matrix $\mathbf{F}^{\mathrm{KS}}$ is calculated as the derivative of the energy with respect to the density matrix. This leads to

$$\begin{aligned} X_{\mu\nu} = &\int \frac{\partial g_{\mathrm{xc}}}{\partial \rho} \mu\nu \ \mathrm{d}^3 r + \int \frac{|\mathbf{j}|^2}{2\rho^2} \frac{\partial g_{\mathrm{xc}}}{\partial \rho} \mu\nu \ \mathrm{d}^3 r \\ &+ \int 2 \frac{\partial g_{\mathrm{xc}}}{\partial \gamma} \nabla n \cdot \left[\{\nabla \mu\} \nu + \mu \{\nabla \nu\}\right] \mathrm{d}^3 r \\ &+ \int \frac{1}{2} \frac{\partial g_{\mathrm{xc}}}{\partial \tilde{\tau}} \left[\{\nabla \mu\} \cdot \{\nabla \nu\}\right] \mathrm{d}^3 r \\ &+ \int \frac{\mathrm{i}}{2} \frac{\mathbf{j}}{\rho} \cdot \frac{\partial g_{\mathrm{xc}}}{\partial \tilde{\tau}} \left[\{\nabla \mu\} \nu - \mu \{\nabla \nu\}\right] \mathrm{d}^3 r \end{aligned} \tag{17}$$

which can be calculated with numerical integration methods as usual.[S10–S14]

In the course of this work, the current-dependent extension was implemented into the RT-TDDFT framework[S5] of the `Riper` module[S15,S16] of TURBOMOLE.[S17–S19] The initial current-independent implementation[S5] uses real algebra exclusively. For the current-dependent metaGGAs, the corresponding complex framework was used for the diagonalization of the Fock matrices and the time evolution operator. We note in passing that the same applies for hybrid functionals. Therefore, an implementation of hybrid DFT can be easily generalized to the current-dependent metaGGA framework *et vice versa.* The required integrals are available from the existing 2c SCF implementation by only considering the particle and z spin (current) densities.

S1.3 Pilot Applications

To demonstrate the consistency of RT-TDDFT and linear response TDDFT with the current-dependent extension, we study a small set of molecules previously studied in ref. S5 at the PBE level. This includes H_2, CO, CH_4, C_6H_6, and H_2O. Structures were optimized in the present work with the def2-SVP orbital[S20] and auxiliary basis set[S21] for the resolution of the identity approximation to the Coulomb integrals (RI-J). The PBE functional[S22] is employed together with a large grid (grid size 5) for the numerical integration.[S10,S23] SCF procedures are converged up to 10^{-8}Hartee for the energy and 10^{-7} for the root mean square of the density matrix change. Structures are listed in the next subsection.

Excitation spectra are then computed with the def2-SVP,[S20] def2-TZVP,[S20] as well as def2-TZVPPD basis set[S24] and the density fitting continuous fast multipole method (DF-CFMM) as implemented in `Riper`.[S15,S16] A Gaussian pulse with an amplitude of $2 \cdot 10^{-5}$ a.u., a width of 0.2 a.u., and t_0 of 3 a.u. was applied. The predictor-correcter scheme and the second-order Magnus expansion are employed with for a total simulation time of 1000 a.u. and a time step of 0.1 a.u.; damping for the dipole strength function was set to 0.004 a.u., see ref. S5 for the detailed definitions. Results with the TPSS,[S25] Tao-Mo (TM),[S26] r^2SCAN,[S27,S28] M06-L,[S29] and MN15-L[S30] functionals are shown in Tables S1–S3.

Overall, the two different formalism show an excellent agreement, The largest impact of the current density is observed with the MN15-L and r^2SCAN functionals and the hydrogen and CO molecule. Here, the impact of the current density is in the same range as the difference between the results of MN15-L and its predecessor M06-L. Notably, the basis set is also of great importance for the results and the excitation energies decrease from def2-SVP to def2-TZVP and def2-TZVPPD. This demonstrates that the current density should also be incorporated in RT-TDDFT studies.

Table S1: Lowest excitation energies in eV with the RT-TDDFT and LR-TDDFT approach for small molecules with metaGGA functionals and the def2-SVP basis set. DFT denotes the current-independent formalism and CDFT refers to the current-dependent ansatz implemented herein.

		TPSS		TM		r^2SCAN		M06-L		MN15-L	
Molecule	Method	DFT	CDFT	DFT	CDFT	DFT	CDFT	DFT	CDFT	DFT	CDFT
H_2	RT	13.49	13.28	12.93	13.13	13.71	13.19	13.64	13.43	13.81	13.16
	LR	13.48	13.28	12.97	13.16	13.71	13.19	13.63	13.43	13.82	13.16
CO	RT	8.47	8.37	8.50	8.41	8.77	8.51	8.58	8.34	9.17	8.63
	LR	8.47	8.37	8.50	8.41	8.77	8.51	8.58	8.34	9.17	8.63
CH_4	RT	11.40	11.37	11.52	11.46	12.11	11.91	12.03	12.02	11.93	11.74
	LR	11.40	11.37	11.52	11.47	12.11	11.94	12.03	12.02	11.93	11.74
C_6H_6	RT	7.22	7.20	7.24	7.22	7.39	7.32	7.30	7.25	7.63	7.53
	LR	7.22	7.21	7.24	7.22	7.39	7.33	7.30	7.25	7.63	7.53
H_2O	RT	7.49	7.46	7.72	7.67	8.54	8.35	8.23	8.16	8.52	8.27
	LR	7.49	7.46	7.72	7.67	8.54	8.35	8.23	8.16	8.52	8.27

Table S2: Lowest excitation energies in eV with the RT-TDDFT and LR-TDDFT approach for small molecules with metaGGA functionals and the def2-TZVP basis set. DFT denotes the current-independent formalism and CDFT refers to the current-dependent ansatz implemented herein.

		TPSS		TM		r^2SCAN		M06-L		MN15-L	
Molecule	Method	DFT	CDFT	DFT	CDFT	DFT	CDFT	DFT	CDFT	DFT	CDFT
H_2	RT	12.90	12.77	12.67	12.64	13.24	12.68	12.96	12.89	12.88	12.42
	LR	12.90	12.78	12.64	12.63	13.24	12.69	12.97	12.90	12.88	12.44
CO	RT	8.41	8.31	8.44	8.35	8.72	8.46	8.56	8.33	9.08	8.57
	LR	8.41	8.31	8.44	8.35	8.72	8.46	8.56	8.34	9.08	8.58
CH_4	RT	10.84	10.81	10.96	10.90	11.59	11.37	11.50	11.50	11.21	11.05
	LR	10.84	10.82	10.96	10.91	11.59	11.40	11.50	11.50	11.21	11.05
C_6H_6	RT	6.99	6.97	7.01	6.99	7.18	7.11	7.10	7.06	7.39	7.30
	LR	6.99	6.97	7.02	6.99	7.18	7.11	7.10	7.06	7.39	7.30
H_2O	RT	7.34	7.32	7.51	7.46	8.22	8.06	7.99	7.95	8.03	7.83
	LR	7.35	7.32	7.51	7.46	8.23	8.07	7.99	7.95	8.03	7.84

Table S3: Lowest excitation energies in eV with the RT-TDDFT and LR-TDDFT approach for small molecules with metaGGA functionals and the def2-TZVPPD basis set. DFT denotes the current-independent formalism and CDFT refers to the current-dependent ansatz implemented herein.

		TPSS		TM		r^2SCAN		M06-L		MN15-L	
Molecule	Method	DFT	CDFT	DFT	CDFT	DFT	CDFT	DFT	CDFT	DFT	CDFT
H_2	RT	12.17	12.09	12.06	12.03	12.87	12.14	12.72	12.68	12.35	12.06
	LR	12.17	12.10	12.05	12.00	12.87	12.18	12.72	12.69	12.35	12.07
CO	RT	8.38	8.29	8.42	8.33	8.70	8.44	8.54	8.32	9.05	8.55
	LR	8.39	8.29	8.42	8.33	8.70	8.44	8.54	8.33	9.05	8.56
CH_4	RT	9.77	9.76	9.95	9.90	10.61	10.43	10.32	10.33	10.07	10.01
	LR	9.77	9.76	9.95	9.91	10.61	10.45	10.32	10.33	10.07	10.00
C_6H_6	RT	6.88	6.87	6.95	6.93	7.13	7.07	7.07	7.03	7.30	7.22
	LR	6.93	6.91	6.96	6.93	7.13	7.07	7.07	7.03	7.30	7.22
H_2O	RT	6.86	6.84	7.03	6.99	7.60	7.48	7.38	7.35	7.42	7.29
	LR	6.86	6.85	7.03	6.99	7.60	7.48	7.39	7.35	7.42	7.29

For completeness, we also demonstrate the consistency of the LR-TTDDFT and RT-TDDFT approach for hybrid functionals using the def2-TZVP basis set. The PBE0,[S31] ωB97X-D,[S32] TPSSh,[S33] and its current-dependent generalization cTPSSh are chosen for this validation. The semilocal PBE[S22] functional is included for comparison. Results are shown in Table S4 and clearly indicate the correctness of the implementation.

Table S4: Lowest excitation energies in eV with the RT-TDDFT and LR-TDDFT approach for small molecules with the PBE, PBE0, ωB97X-D, TPSSh, and cTPSSh functionals and the def2-TZVP basis set.

		PBE	PBE0	ωB97X-D	TPSSh	cTPSSh
H_2	RT	12.49	12.76	12.66	12.96	12.84
	LR	12.49	12.76	12.66	12.96	12.85
CO	RT	8.21	8.35	8.39	6.45	8.36
	LR	8.21	8.35	8.39	8.45	8.36
CH_4	RT	10.46	11.04	11.25	11.04	11.01
	LR	10.46	11.04	11.25	11.03	11.01
C_6H_6	RT	6.88	7.05	7.07	7.05	7.03
	LR	6.88	7.05	7.07	7.05	7.03
H_2O	RT	7.05	7.66	7.84	7.56	7.54
	LR	7.05	7.66	7.84	7.56	7.54

S1.4 Molecular Structures

Optimized structures at the def2-SVP/PBE/RI-J level of theory (grid size 5).[S10,S20–S23] SCF energies converged to at least 10^{-8} Hartree and 10^{-7} a.u. for the change of the density matrix based on the root mean square. Point-group symmetry was exploited in the structure optimizations. Structures are given in Ångstrom. Energies are given in Hartree.

```
Hydrogen Molecule
2
Energy = -1.16068636997
H     0.0000000    0.0000000    0.3840607
H     0.0000000    0.0000000   -0.3840607
```

```
Carbon Monoxide
2
Energy = -113.09947432769
C    -0.3062194    0.0000000    0.0000000
O     0.8353966    0.0000000    0.0000000
```

```
Methane
5
Energy = -40.41519131809
C     0.0000000    0.0000000    0.0000000
H     0.6385179   -0.6385179    0.6385179
H    -0.6385179    0.6385179    0.6385179
H    -0.6385179   -0.6385179   -0.6385179
H     0.6385179    0.6385179   -0.6385179
```

```
Water
3
Energy = -76.27267183447
O     0.0000000    0.0000000    0.4082914
H    -0.7581074    0.0000000   -0.2041463
H     0.7581074    0.0000000   -0.2041463
```

Benzene

12

Energy = -231.77414209618

C	1.2165544	-0.7023780	0.0000000
C	1.2165544	0.7023780	0.0000000
C	-0.0000000	1.4047560	0.0000000
C	-1.2165544	0.7023780	0.0000000
C	-1.2165544	-0.7023780	0.0000000
C	-0.0000000	-1.4047560	0.0000000
H	2.1701661	-1.2529460	0.0000000
H	2.1701661	1.2529460	0.0000000
H	-0.0000000	2.5058920	0.0000000
H	-2.1701661	1.2529460	0.0000000
H	-2.1701661	-1.2529460	0.0000000
H	-0.0000000	-2.5058920	0.0000000

References

(S1) Goings, J. J.; Lestrange, P. J.; Li, X. Real-time time-dependent electronic structure theory. *Wiley Interdiscip. Rev.: Comput. Mol. Sci.* **2018**, *8*, e1341, DOI: `https://doi.org/10.1002/wcms.1341`.

(S2) Li, X.; Govind, N.; Isborn, C.; DePrince, A. E. I.; Lopata, K. Real-Time Time-Dependent Electronic Structure Theory. *Chem. Rev.* **2020**, *120*, 9951–9993, DOI: `10.1021/acs.chemrev.0c00223`.

(S3) Magnus, W. On the exponential solution of differential equations for a linear operator. *Comm. Pure Appl. Math.* **1954**, *7*, 649–673, DOI: `https://doi.org/10.1002/cpa.3160070404`.

(S4) Blanes, S.; Casas, F.; Oteo, J.; Ros, J. The Magnus expansion and some of its applications. *Phys. Rep.* **2009**, *470*, 151–238, DOI: `https://doi.org/10.1016/j.physrep.2008.11.001`.

(S5) Müller, C.; Sharma, M.; Sierka, M. Real-time time-dependent density functional theory using density fitting and the continuous fast multipole method. *J. Comput. Chem.* **2020**, *41*, 2573–2582, DOI: `https://doi.org/10.1002/jcc.26412`.

(S6) Wu, X.; Teuler, J.-M.; Cailliez, F.; Clavaguéra, C.; Salahub, D. R.; de la Lande, A. Simulating Electron Dynamics in Polarizable Environments. *J. Chem. Theory Comput.* **2017**, *13*, 3985–4002, DOI: `10.1021/acs.jctc.7b00251`.

(S7) Cheng, C.-L.; Evans, J. S.; Van Voorhis, T. Simulating molecular conductance using real-time density functional theory. *Phys. Rev. B* **2006**, *74*, 155112, DOI: `10.1103/PhysRevB.74.155112`.

(S8) Lopata, K.; Govind, N. Modeling Fast Electron Dynamics with Real-Time Time-Dependent Density Functional Theory: Application to Small Molecules and Chromophores. *J. Chem. Theory Comput.* **2011**, *7*, 1344–1355, DOI: `10.1021/ct200137z`.

(S9) Morzan, U. N.; Ramírez, F. F.; Oviedo, M. B.; Sánchez, C. G.; Scherlis, D. A.; Lebrero, M. C. G. Electron dynamics in complex environments with real-time time dependent density functional theory in a QM-MM framework. *J. Chem. Phys.* **2014**, *140*, 164105, DOI: `10.1063/1.4871688`.

(S10) Treutler, O.; Ahlrichs, R. Efficient molecular numerical integration schemes. *J. Chem. Phys.* **1995**, *102*, 346–354, DOI: `10.1063/1.469408`.

(S11) Gill, P. M.; Johnson, B. G.; Pople, J. A. A standard grid for density functional calculations. *Chem. Phys. Lett.* **1993**, *209*, 506–512, DOI: `10.1016/0009-2614(93)80125-9`.

(S12) Stratmann, R.; Scuseria, G. E.; Frisch, M. J. Achieving linear scaling in exchange-correlation density functional quadratures. *Chem. Phys. Lett.* **1996**, *257*, 213–223, DOI: `10.1016/0009-2614(96)00600-8`.

(S13) Bates, J. E.; Furche, F. Harnessing the meta-generalized gradient approximation for time-dependent density functional theory. *J. Chem. Phys.* **2012**, *137*, 164105, DOI: 10.1063/1.4759080.

(S14) Holzer, C.; Franzke, Y. J.; Pausch, A. Current density functional framework for spin–orbit coupling. *J. Chem. Phys.* **2022**, *157*, 204102, DOI: 10.1063/5.0122394.

(S15) Burow, A. M.; Sierka, M.; Mohamed, F. Resolution of identity approximation for the Coulomb term in molecular and periodic systems. *J. Chem. Phys.* **2009**, *131*, 214101, DOI: 10.1063/1.3267858.

(S16) Burow, A. M.; Sierka, M. Linear scaling hierarchical integration scheme for the exchange-correlation term in molecular and periodic systems. *J. Chem. Theory Comput.* **2011**, *7*, 3097–3104, DOI: 10.1021/ct200412r.

(S17) Balasubramani, S. G.; Chen, G. P.; Coriani, S.; Diedenhofen, M.; Frank, M. S.; Franzke, Y. J.; Furche, F.; Grotjahn, R.; Harding, M. E.; Hättig, C.; Hellweg, A.; Helmich-Paris, B.; Holzer, C.; Huniar, U.; Kaupp, M.; Marefat Khah, A.; Karbalaei Khani, S.; Müller, T.; Mack, F.; Nguyen, B. D.; Parker, S. M.; Perlt, E.; Rappoport, D.; Reiter, K.; Roy, S.; Rückert, M.; Schmitz, G.; Sierka, M.; Tapavicza, E.; Tew, D. P.; van Wüllen, C.; Voora, V. K.; Weigend, F.; Wodyński, A.; Yu, J. M. TURBOMOLE: Modular program suite for *ab initio* quantum-chemical and condensed-matter simulations. *J. Chem. Phys.* **2020**, *152*, 184107, DOI: 10.1063/5.0004635.

(S18) Franzke, Y. J.; Holzer, C.; Andersen, J. H.; Begušić, T.; Bruder, F.; Coriani, S.; Della Sala, F.; Fabiano, E.; Fedotov, D. A.; Fürst, S.; Gillhuber, S.; Grotjahn, R.; Kaupp, M.; Kehry, M.; Krstić, M.; Mack, F.; Majumdar, S.; Nguyen, B. D.; Parker, S. M.; Pauly, F.; Pausch, A.; Perlt, E.; Phun, G. S.; Rajabi, A.; Rappoport, D.; Samal, B.; Schrader, T.; Sharma, M.; Tapavicza, E.; Treß, R. S.; Voora, V.; Wodyński, A.; Yu, J. M.; Zerulla, B.; Furche, F.; Hättig, C.; Sierka, M.; Tew, D. P.; Weigend, F. TURBOMOLE: Today and Tomorrow. *J. Chem. Theory Comput.* **2023**, *19*, 6859–6890, DOI: 10.1021/acs.jctc.3c00347.

(S19) TURBOMOLE GmbH 2024; Developers' version of TURBOMOLE V7.9, a development of University of Karlsruhe and Forschungszentrum Karlsruhe GmbH, 1989-2007, TURBOMOLE GmbH, since 2007; available from https://www.turbomole.org (retrieved December 9, 2024).

(S20) Weigend, F.; Ahlrichs, R. Balanced basis sets of split valence, triple zeta valence and quadruple zeta valence quality for H to Rn: Design and assessment of accuracy. *Phys. Chem. Chem. Phys.* **2005**, *7*, 3297–3305, DOI: 10.1039/B508541A.

(S21) Weigend, F. Accurate Coulomb-fitting basis sets for H to Rn. *Phys. Chem. Chem. Phys.* **2006**, *8*, 1057–1065, DOI: 10.1039/B515623H.

(S22) Perdew, J. P.; Burke, K.; Ernzerhof, M. Generalized Gradient Approximation Made Simple. *Phys. Rev. Lett.* **1996**, *77*, 3865–3868, DOI: 10.1103/PhysRevLett.77.3865.

(S23) Treutler, O. Entwicklung und Anwendung von Dichtefunktionalmethoden. Dissertation (Dr. rer. nat.), University of Karlsruhe (TH), Germany, 1995.

(S24) Rappoport, D.; Furche, F. Property-optimized Gaussian basis sets for molecular response calculations. *J. Chem. Phys.* **2010**, *133*, 134105, DOI: 10.1063/1.3484283.

(S25) Tao, J.; Perdew, J. P.; Staroverov, V. N.; Scuseria, G. E. Climbing the Density Functional Ladder: Nonempirical Meta–Generalized Gradient Approximation Designed for Molecules and Solids. *Phys. Rev. Lett.* **2003**, *91*, 146401, DOI: 10.1103/PhysRevLett.91.146401.

(S26) Tao, J.; Mo, Y. Accurate Semilocal Density Functional for Condensed-Matter Physics and Quantum Chemistry. *Phys. Rev. Lett.* **2016**, *117*, 073001, DOI: 10.1103/PhysRevLett.117.073001.

(S27) Furness, J. W.; Kaplan, A. D.; Ning, J.; Perdew, J. P.; Sun, J. Accurate and Numerically Efficient r^2SCAN Meta-Generalized Gradient Approximation. *J. Phys. Chem. Lett.* **2020**, *11*, 8208–8215, DOI: 10.1021/acs.jpclett.0c02405.

(S28) Furness, J. W.; Kaplan, A. D.; Ning, J.; Perdew, J. P.; Sun, J. Correction to "Accurate and Numerically Efficient r^2SCAN Meta-Generalized Gradient Approximation". *J. Phys. Chem. Lett.* **2020**, *11*, 9248–9248, DOI: 10.1021/acs.jpclett.0c03077.

(S29) Zhao, Y.; Truhlar, D. G. A new local density functional for main-group thermochemistry, transition metal bonding, thermochemical kinetics, and noncovalent interactions. *J. Chem. Phys.* **2006**, *125*, 194101, DOI: 10.1063/1.2370993.

(S30) Yu, H. S.; He, X.; Truhlar, D. G. MN15-L: A New Local Exchange-Correlation Functional for Kohn–Sham Density Functional Theory with Broad Accuracy for Atoms, Molecules, and Solids. *J. Chem. Theory Comput.* **2016**, *12*, 1280–1293, DOI: 10.1021/acs.jctc.5b01082.

(S31) Adamo, C.; Barone, V. Toward reliable density functional methods without adjustable parameters: The PBE0 model. *J. Chem. Phys.* **1999**, *110*, 6158–6170, DOI: 10.1063/1.478522.

(S32) Chai, J.-D.; Head-Gordon, M. Long-range corrected hybrid density functionals with damped atom–atom dispersion corrections. *Phys. Chem. Chem. Phys.* **2008**, *10*, 6615–6620, DOI: 10.1039/B810189B.

(S33) Staroverov, V. N.; Scuseria, G. E.; Tao, J.; Perdew, J. P. Comparative assessment of a new nonempirical density functional: Molecules and hydrogen-bonded complexes. *J. Chem. Phys.* **2003**, *119*, 12129–12137, DOI: 10.1063/1.1626543.
